# Implementation strategies for integrating non-communicable disease care into primary healthcare settings in sub-Saharan Africa: A systematic review

**DOI:** 10.1371/journal.pgph.0007062

**Published:** 2026-09-15

**Authors:** Hubert Amu, Theodora Yayra Brinsley, Joshua Oppong

**Affiliations:** 1 Department of Population and Behavioural Sciences, Fred N. Binka School of Public Health, University of Health and Allied Sciences, Hohoe, Ghana; 2 Department of Epidemiology and Disease Control, School of Public Health, University of Ghana, Accra, Ghana; African Vision Research Institute, SOUTH AFRICA

## Abstract

This sytematic review synthesised evidence on implementation strategies for integrating non-communicable disease (NCD) care into primary healthcare (PHC) in Sub-Saharan Africa (SSA), the barriers and facilitators influencing implementation, the effects on service delivery and patient outcomes, and implications for scale-up and sustainability. Our review followed PRISMA 2020 guidelines using the Population–Exposure–Outcome (PEO) framework. Studies were identified through searches of PubMed/MEDLINE, African Journals Online, and Google Scholar. We included English-language studies published between 1 January 1990 and 31 December 2025. Methodological quality was appraised using the Mixed Methods Appraisal Tool (MMAT). Narrative synthesis followed the Synthesis Without Meta-analysis (SWiM) guideline. From 22,959 records initially identified, 46 studies met the inclusion criteria. The Studies were conducted in 14 SSA countries. Six implementation-strategy clusters were identified: task-shifting and task-sharing; integrated chronic care models; workforce capacity-building, training and educational outreach; digital health and decision-support tools; community- and group-based delivery; and guidelines, protocols, and quality-improvement packages. Barriers to implementation included workforce shortages, supply-chain interruptions, weak guideline operationalisation, and limited digital infrastructure. Engaged frontline leadership, supportive organisational climate, integration with existing HIV chronic care platforms, drug revolving funds, and community partnerships were the most consistent facilitators. Implementation strategies were associated with improvements in service delivery, provider knowledge and competence, and selected clinical outcomes (notably blood pressure control), but effects on diabetes control, retention, and longer-term sustainability were less consistent. Implementation strategies that integrate NCD care into PHC in SSA can deliver meaningful gains in access, provider capability, and clinical control, but their effects depend on health-system foundations and sustained leadership. Scaling up integration will require investment in workforce, supply chains, and information systems, embedded leadership, alignment with existing HIV chronic care platforms, and iterative, context-adapted design. These priorities are central to progress towards Sustainable Development Goals 3.4 and 3.8.

## Background

Non-communicable diseases (NCDs) are the leading cause of mortality, accounting for three-quarters of all deaths globally [1]. Low- and middle-income countries (LMICs), especially in sub-Saharan Africa (SSA), are increasingly affected by this burden due to rapid urbanisation, demographic shifts, and lifestyle changes that contribute to the growing prevalence of chronic disease [2,3]. SSA is consequently experiencing a complex epidemiological transition characterised by a persistent burden of infectious diseases alongside a rapidly increasing burden of NCDs, placing additional pressure on already resource-constrained health systems.

Health systems in SSA have traditionally been organised around vertical, disease-specific programmes, often supported by external funding directed towards communicable diseases such as HIV/AIDS, tuberculosis, and malaria [4]. These programmes have played an important role in strengthening public health. However, they have also contributed to fragmented service delivery structures that are not well-suited to the long-term and coordinated care required for NCD management. Consequently, individuals living with chronic conditions often experience challenges, such as limited access to essential diagnostics and medications, poor continuity of care, weak referral systems, and inadequate follow-up, particularly at the primary healthcare (PHC) level [5].

PHC in SSA is typically organised as a tiered, decentralised system, in which community-level health posts and health centres provide first-contact care and refer onward to district hospitals, supported at the community level by cadres of community health workers. Frontline services are delivered predominantly by nurses, midwives, clinical or medical officers, and other non-physician providers, with physicians concentrated at higher levels of the system and often scarce at the primary level [6]. Historically, these facilities have prioritised maternal and child health, immunisation, and communicable-disease programmes frequently delivered through dedicated, vertically funded platforms [4]. Service readiness for chronic non-communicable disease care remains uneven: many facilities contend with shortages of trained staff, intermittent availability of essential medicines and diagnostics, limited laboratory and information systems, and constrained infrastructure, all of which shape the feasibility of delivering continuous NCD care [7]. These structural features form the basis on which strategies to integrate NCD care into PHC are designed, and they help explain both the opportunities for, and the constraints on, the integration examined in this review.

PHC provides a critical platform for delivering comprehensive and accessible health services. The 2018 Declaration of Astana reaffirmed global commitment to PHC as the foundation for achieving Universal Health Coverage (UHC) [8]. This commitment emphasises the importance of integrated, people-centred, and continuous care across the life course. Integration of NCD services into PHC systems has been identified as a key strategy for strengthening health systems, improving accessibility, and enhancing health outcomes in LMICs. The World Health Organization (WHO) has reinforced this approach through the Package of Essential Noncommunicable Disease Interventions (WHO PEN), which promotes the delivery of cost-effective NCD services at the primary care level [9].

Despite the strong policy endorsements, progress in integrating NCD care into PHC across SSA has been uneven. Differences in health system capacity, workforce availability, infrastructure, governance, and financing mechanisms continue to influence the extent and effectiveness of integration efforts [7]. Various implementation strategies have been adopted to support integration. These include task-shifting, decentralisation of services to enhance access, adoption of integrated chronic care models, utilisation of digital health technologies such as mobile health (mHealth) and telemedicine, and strengthening of referral and supply chain systems [6,10]. However, the effectiveness of these strategies is highly context-dependent and varies across geographic locations.

The evidence base on implementation strategies for integrating NCD care into PHC in SSA remains fragmented and heterogeneous. Existing studies vary in design, scope, intervention types, and outcome measures [11,12]. These include quantitative evaluations of service delivery and clinical outcomes, qualitative studies examining implementation processes and contextual factors, and mixed-methods studies that integrate both perspectives. Although this methodological diversity provides insights, it also presents challenges for synthesis and limits the ability of policymakers and practitioners to identify implementation strategies that are effective, scalable, and sustainable across different contexts.

Integrating NCD care into PHC also aligns with global development priorities, specifically SDG 3.4, which seeks to reduce premature mortality from NCDs through prevention and treatment, and SDG 3.8, which aims to achieve UHC through access to quality essential healthcare services and affordable medicines [13]. Strengthening PHC systems to deliver integrated NCD services may further contribute to reducing health-related financial hardship (SDG 1), addressing inequalities in access to healthcare (SDG 10), and strengthening partnerships for health system development (SDG 17) [13].

Given the growing burden of NCDs and the central role of PHC in addressing it, there is a need to synthesise evidence on how NCD integration is being implemented across SSA and which strategies are most effective in different contexts. Although previous reviews have examined specific models of care and interventions [14,15], there is a gap in systematically analysing implementation strategies, including the strategies used, the barriers and facilitators influencing their implementation, the reported effects on service delivery and patient outcomes, and the implications for scale-up and sustainability.

To address this gap, the present review posed four questions: (i) which implementation strategies are used to integrate NCD care into PHC in SSA; (ii) what barriers and facilitators influence their implementation; (iii) what effects are reported on service delivery and patient outcomes; and (iv) what implications arise for scale-up, sustainability, and health-system strengthening.

## Methods

### Study design and methodological standards

Our review synthesised qualitative, quantitative, and mixed-methods evidence on implementation strategies for integrating NCD care into PHC in SSA. The review was reported in accordance with the Preferred Reporting Items for Systematic Reviews and Meta-Analyses (PRISMA) 2020 guidelines [16] (S1 Checklist). The protocol was developed following the Preferred Reporting Items for Systematic Review and Meta-Analysis Protocols (PRISMA-P) 2015 statement to ensure transparency and reproducibility [17] and was registered with the International Prospective Register of Systematic Reviews (PROSPERO: CRD420261354803). Due to the heterogeneity in study designs, intervention types, and outcome measures, narrative synthesis was the primary synthesis method adopted, following the Synthesis Without Meta-analysis (SWiM) guideline [18]. Our review process (literature searching, study selection, data extraction, and synthesis) was guided by the Joanna Briggs Institute (JBI) Manual for Evidence Synthesis [19].

### Implementation strategies

Implementation strategies are defined as the methods or techniques used to enhance the adoption, implementation, sustainment, and scale-up of an intervention [20]. In this review, they denote the approaches used to integrate NCD care into PHC (task-shifting, integrated care models, workforce training, digital tools, community-based delivery, and guideline or quality-improvement packages) as distinct from the clinical interventions themselves.

### Eligibility criteria

#### Geographic scope, time window, and language.

We included studies that were conducted in SSA, defined by the World Bank regional classification [21]. The search covered studies published in English between 1 January 1990 and 31 December 2025. This period was chosen to capture both early and contemporary integration efforts. Our review was restricted to English-language publications, which may have introduced language bias against Francophone and Lusophone evidence; this is acknowledged as a limitation.

#### PEO framework.

Eligibility criteria were defined using the Population–Exposure–Outcome (PEO) framework, which is well-suited to reviews of implementation evidence and enables the systematic inclusion of diverse study designs [19].

**Population:** Our population included studies conducted among individuals receiving NCD-related services, including prevention, screening, diagnosis, treatment, follow-up, and chronic care within PHC settings. It also included health workers and PHC teams involved in delivering NCD care, including medical doctors, nurses, midwives, community health workers (CHWs), physician assistants, and other non-physician providers.

**Exposure:** The exposure of interest was implementation strategies or delivery models aimed at integrating NCD care into PHC. Strategies included task-shifting and task-sharing, decentralisation of services, integrated clinic models, implementation of clinical guidelines and protocols, workforce strengthening interventions, referral pathway strengthening, digital health and decision-support tools (mHealth, telemedicine), and community-based PHC programmes targeting NCDs. Where data permitted, implementation strategies were described using recommended reporting dimensions (actor, action, target, temporality, dose). The Expert Recommendations for Implementing Change (ERIC) taxonomy was used as a reference to standardise the classification of strategies where feasible [22].

**Outcomes:** Studies were eligible if they reported on the implementation, application, or evaluation of at least one strategy for integrating NCD care into PHC settings in SSA. The primary outcome of interest was the implementation strategy itself, characterised by its description, actors, actions, setting, and duration of application. Secondary outcomes, extracted where reported but not required for inclusion, comprised implementation outcomes (acceptability, adoption, appropriateness, feasibility, fidelity, cost, penetration, and sustainability); service-delivery outcomes (access, coverage, continuity of care, quality indicators, adherence to guidelines, referral completion, and supply-chain performance); and patient-level clinical outcomes (blood-pressure control, glycaemic control, retention in care, treatment adherence, complication rates, and patient experience). Studies focused solely on NCD epidemiology or clinical effectiveness without describing an implementation or integration strategy were excluded.

### Types of studies

We included a broad range of empirical study designs, including randomised controlled trials and quasi-experimental studies, observational studies (cross-sectional, cohort, interrupted time series), qualitative studies exploring implementation experiences, barriers, facilitators, acceptability, and feasibility, and mixed-methods studies. Studies were excluded if they were conducted exclusively in secondary or tertiary care facilities without a PHC integration component, focused solely on NCD epidemiology or clinical effectiveness without an implementation or integration strategy, or were editorials, commentaries, or opinion pieces. A summary of the eligibility criteria is provided in S1 Table.

### Information sources and search strategy

Electronic searches were conducted in PubMed/MEDLINE, African Journals Online (AJOL), and Google Scholar. Reference lists of all included studies were manually screened, and forward citation tracking was conducted in Google Scholar. PubMed/MEDLINE was selected for comprehensive indexing of the international biomedical literature; AJOL was included specifically to capture African-published journals, thereby improving the representation of regionally generated evidence for a review focused on sub-Saharan Africa. Our search strategy combined controlled vocabulary (Medical Subject Headings [MeSH]) with free-text keywords across four concept blocks derived from the PEO framework: (i) non-communicable diseases (including general and condition-specific terminology, hypertension, diabetes, cardiovascular disease, stroke, cancer, chronic respiratory disease, asthma, COPD); (ii) primary healthcare (primary care, community health services, family practice); (iii) integration and implementation strategies (integrated care, service integration, implementation strategy, task-shifting/sharing, decentralisation, guideline implementation, quality improvement, digital health, mHealth, telemedicine); and (iv) sub-Saharan Africa (regional and country-specific terms). Within each block, terms were combined using the Boolean operator OR; the four blocks were combined using AND. Truncation and phrase searching were used as appropriate (S1 Text).

### Study selection

Study selection was conducted in two independent stages. In the first stage, TYB and JO independently screened titles and abstracts against a standardised screening form. In the second stage, the full texts of all potentially eligible studies were independently assessed against the eligibility criteria by the two reviewers. Any disagreements arising at either stage were resolved through discussion and consensus; where consensus could not be reached, HA was consulted for arbitration. Decisions were documented, and reasons for exclusion at the full-text stage were also recorded to support the construction of the PRISMA 2020 flow diagram. All identified records were imported into the reference management software Zotero for de-duplication.

### Data extraction

We used a standardised data extraction form, which was developed from the JBI Manual and piloted on a subset of included studies before full extraction. The fields comprised bibliographic details (author, year, country, publication type); study characteristics (aims, design, sampling, PHC setting); the NCD conditions addressed; detailed descriptions of the integration model and implementation strategies, including who implemented the strategy, what actions were undertaken, where they were implemented, and over what period; reported barriers and facilitators to implementation; and outcomes reported, including measurement tools and time points. Data extraction was conducted independently by two reviewers, with discrepancies resolved through discussion (S2 Table).

### Handling of missing and incomplete data

As this review synthesised evidence from previously published studies, participant-level missing data were not applicable. Where study reports contained incomplete or unclear information, the two reviewers examined the full-text articles and any accompanying supplementary materials to clarify the reported methods or findings. No study authors were contacted for additional information, and no data were obtained from sources outside the published reports.

### Quality appraisal

We assessed the methodological quality of the included studies using the Mixed Methods Appraisal Tool (MMAT), version 2018 [23]. We evaluated each study against the five MMAT criteria relevant to its design. The assessments were conducted independently by JO and TYB. Results were presented item-by-item in evidence tables (S3 Table). A composite score was not computed to ensure transparent reporting of methodological limitations. An overall confidence rating of high, moderate, or low was assigned to each study to support synthesis.

### Data synthesis

Narrative synthesis was performed in accordance with the SWiM reporting guideline [18]. The synthesis was structured around four thematic groupings derived from the review objectives: (1) the implementation strategies used to integrate NCD care into PHC; (2) the barriers and facilitators that shape their implementation; (3) the reported effects on service delivery and patient outcomes; and (4) the implications for scale-up, sustainability, and health system strengthening. Within each main theme, findings were tabulated and compared across studies to identify recurring patterns and convergent or divergent findings, and to examine how these related to study context (country, PHC setting, NCD condition, and design). Implementation strategies were grouped inductively into clusters and cross-checked against the ERIC taxonomy (Table 2). Barriers and facilitators were organised at the individual, organisational, and system levels, consistent with established implementation science perspectives [24,25]. To strengthen alignment with established implementation-science taxonomies, the strategies identified in each study were mapped to discrete strategies within the ERIC compilation [26], and the six clusters used to organise the synthesis were characterised by their most representative ERIC strategies and categories (Table 2). Where studies reported sufficient detail, strategies were additionally specified using the reporting dimensions recommended by Proctor et al. (actor, action, action target, temporality, and dose) [20]. Quantitative findings were summarised descriptively and integrated narratively with qualitative findings. No statistical pooling was undertaken, given heterogeneity in interventions, populations, and outcome measures.

### Assessment of publication bias and certainty of evidence

Due to the substantial heterogeneity in study designs, interventions, outcomes, and the absence of quantitative synthesis, a formal assessment of publication bias was not undertaken. The certainty of the evidence was graded narratively for the main thematic findings, using the principles from the GRADE approach for quantitative findings [27] and GRADE-CERQual for qualitative findings [28], based on study quality, consistency, directness, and relevance to the review questions. A summary of the certainty assessments is provided in S4 Table.

### Ethics and dissemination

Ethical approval was not required, as the review used only previously published, publicly available data. The protocol was registered in PROSPERO (CRD420261354803) and reported in accordance with PRISMA-P guidelines.

### Protocol deviations

Several deviations from the review protocol were made during its conduct. First, although the protocol specified searches of PubMed/MEDLINE, Scopus, Web of Science, African Journals Online (AJOL), and Google Scholar, the final review searched PubMed/MEDLINE, AJOL, and Google Scholar only. Searches of Scopus and Web of Science were not undertaken because of institutional access constraints. To minimise the potential impact on study identification, Google Scholar was searched comprehensively alongside the other databases. Second, whereas the protocol planned to include both peer-reviewed and grey literature, the final review was restricted to peer-reviewed publications. This decision was made to ensure that all included evidence had undergone external scientific review and to improve the methodological consistency of the evidence base.

## Results

### Study selection

**Fig 1:**
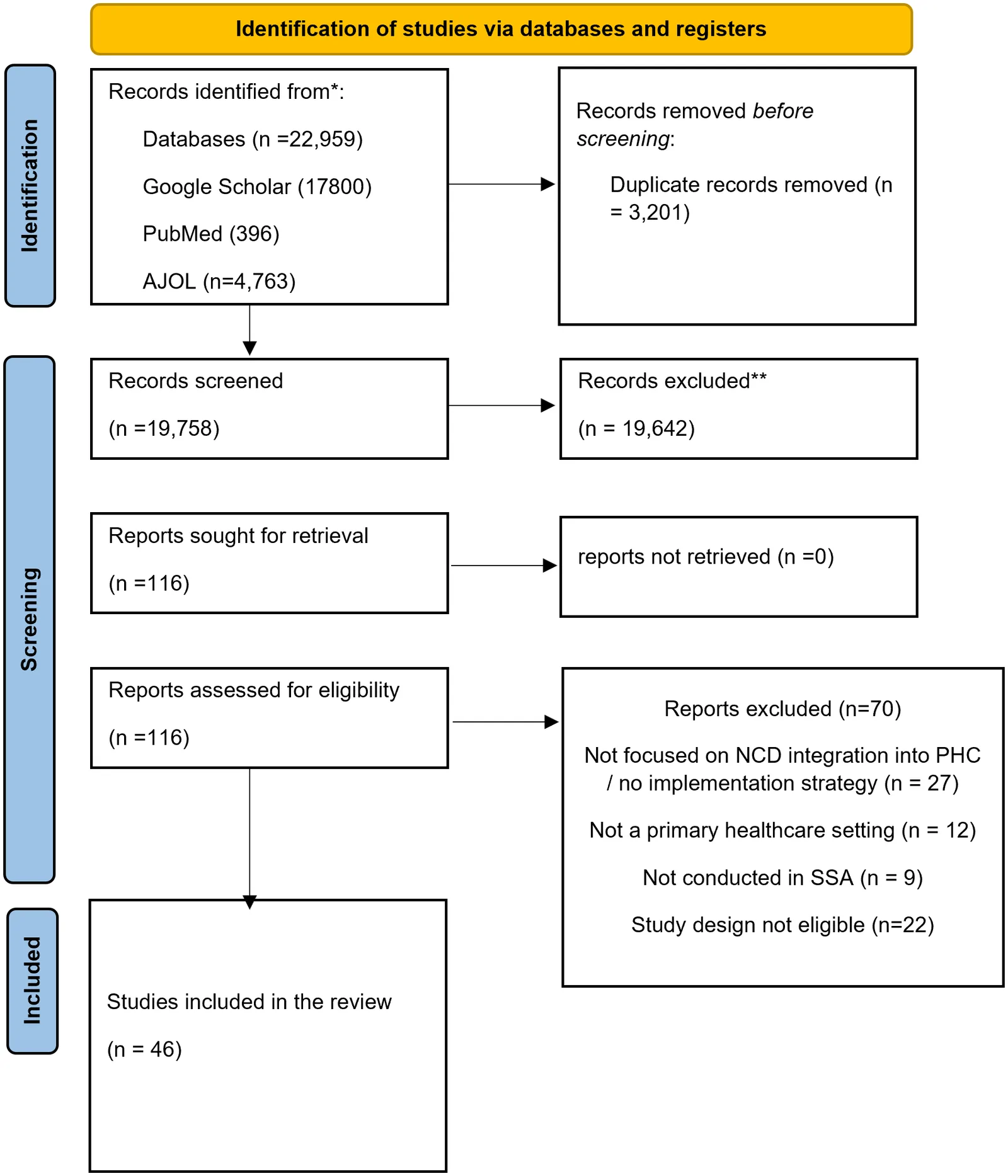
PRISMA 2020 flow diagram.

A total of 22,959 records were identified from electronic database searches. After the removal of 3,201 duplicate records, 19,758 were screened by title and abstract, of which 116 reports were assessed for full-text eligibility. Following full-text screening, 70 reports were excluded, with the most frequent reasons being: not focused on NCD integration into PHC or no implementation strategy described (n = 27), study design not eligible (n = 22), not a PHC setting (n = 12), and not conducted in SSA (n = 9). Consequently, 46 studies met the predefined inclusion criteria for implementation strategies for integrating NCD care into PHC in SSA and were included in this systematic review. The PRISMA flow diagram, which summarises the study identification, screening, eligibility, and inclusion process, is presented in Fig 1.

### Study characteristics

Forty-six studies were included in the final synthesis. Geographically, the evidence base was distributed across 14 SSA countries. South Africa contributed the largest share of studies (n = 19), followed by Nigeria (n = 5), Zimbabwe (n = 4), Uganda (n = 4), Malawi (n = 2), Ethiopia (n = 2), Kenya (n = 2), Ghana (n = 2), Cameroon (n = 1), Mozambique (n = 1), Burkina Faso (n = 1), Eswatini (n = 1), Zambia (n = 1), and the Democratic Republic of the Congo (DRC) (n = 1). Studies were conducted in a range of PHC settings, including community health centres, district-level PHC clinics, integrated chronic disease management facilities, HIV-NCD integrated care platforms, emergency primary care, mobile and telehealth-based services, and community-based pension delivery points.

Study designs spanned the full spectrum permitted under the PEO framework. Quantitative descriptive and non-randomised designs predominated, including cross-sectional surveys, retrospective cohort studies, before-after evaluations, and operational implementation studies; one pragmatic cluster randomised controlled trial [29] and one feasibility individually randomised trial [30] were included; one type 2 hybrid effectiveness-implementation interrupted time series trial [31] and one hybrid-effectiveness study [32] further strengthened the implementation evidence base; the remainder included qualitative studies, mixed-methods studies, and methodologically novel designs such as system dynamics modelling [33], human-centred design [34], and theory of change workshops [35].

Sample sizes varied widely, from small qualitative studies of nine staff members [36] and 10 nurses [37] through pilot evaluations of 20 participants [38], to large facility-level evaluations of 4,393 patients across 38 clinics [29], 6,233 patients across 14 facilities [39], and 21,922 patients with hypertension across 60 PHCs [31]. NCD conditions addressed included hypertension and diabetes (most frequently, often in combination), cardiovascular disease and risk factors more broadly, cervical and breast cancer, diabetic retinopathy, depression and other mental health conditions in chronic care, gestational diabetes, and HIV-NCD multimorbidity. Implementation strategies addressed in the included studies covered task-shifting and task-sharing to nurses, CHWs, and non-physician clinicians; integrated chronic care models; workforce capacity-building, training, and educational outreach; digital health and decision-support tools; community- and group-based delivery models; and the implementation of guidelines, protocols, and quality-improvement packages. Detailed characteristics of the included studies are presented in Table 1.

**Table 1:** Characteristics of the included studies.

Citation	Year	Country	Study Design	Sample Size	Population/ Setting	Strategy/ Focus
Lebina et al. [40]	2020	South Africa	Cross-sectional mixed methods	30 HCWs; 4 PHC facilities	HCWs implementing the ICDM model in two health districts	Integrated chronic disease management model — implementation fidelity
Simelane et al. [41]	2025	South Africa	Qualitative (IDIs)	31 (18 HCPs, 13 PLWH+HTN)	Six PHC facilities, Johannesburg	Patient education for hypertension self-management (iHEART-SA)
Draper et al. [42]	2014	South Africa	Qualitative case study	10 IDIs; 8 FGDs	One comprehensive PHC facility, Cape Town	Alignment between chronic disease policy and practice
Bekele et al. [43]	2024	Ethiopia	Mixed methods (convergent-parallel)	11 interviews + 124 ORIC respondents; 18 facilities	PHC clinicians and administrators, southern Ethiopia	Implementation of Ethiopian PHC Clinical Guidelines for NCDs and mental health
Kamvura et al. [37]	2022	Zimbabwe	Qualitative (IDIs)	10 nurses; 5 urban clinics	General nurses in PHC facilities	Barriers to NCD care provision (HTN, DM, depression)
Jatho et al. [44]	2020	Uganda	Implementation/capacity-building with follow-up	488 PHC workers; 118 districts	PHC workers across 118 districts	National cancer prevention and early detection capacity-building
Ye et al. [33]	2024	Nigeria	System dynamics modelling (CBPR)	16 stakeholders	Hypertension care stakeholders in Nigerian PHC	CBPR + system dynamics for HTN retention
Ratnayake et al. [45]	2021	DR Congo	Cohort with mixed-methods evaluation	833 patients	Emergency PHC facilities, Beni Region (conflict-affected)	NCD integration into emergency primary care
Fairall et al. [29]	2016	South Africa	Pragmatic cluster RCT	4,393 patients; 38 clinics	Patients with HTN, DM, respiratory disease, depression	Educational outreach + integrated clinical management tool (PC101)
Harris et al. [46]	2022	Mozambique	Implementation with pre-post evaluation	60 PHC workers; 11 trainers	Frontline PHC workers, Maputo	Cascade Training-of-Trainers for cardiovascular risk
Asgary et al. [47]	2020	Eswatini	Cohort study	4,247 patients screened	Women in rural Shiselweni region	Smartphone-based mHealth mentorship for cervical cancer screening (VIA)
Saban et al. [48]	2025	South Africa	Convergent mixed methods	35 patients in 4 groups + HCWs	Type 2 diabetes patients at Grassy Park CDC, Cape Town	Group Empowerment and Training (GREAT) for type 2 diabetes
Slingers & De Villiers [49]	2009	South Africa	Before–after study	200 patients (100 + 100)	Hypertension club at Mitchell’s Plain CHC, Cape Town	Hypertension club for HTN management
Wroe et al. [39]	2020	Malawi	Retrospective cohort	6,233 patients; 14 facilities	HIV and NCD patients, Neno district, rural Malawi	Integrated Chronic Care Clinic (IC3) — HIV-NCD integration
Leung et al. [34]	2020	Kenya	Human-centred design + feasibility pilot	Design team + pilot participants	NCD patients and community actors, rural Western Kenya	Human-centred design for NCD care delivery (BIGPIC)
Mensah et al. [32]	2021	Burkina Faso	Hybrid effectiveness (type 2)	350 women	Two PHC centres, Ouagadougou	HPV-based cervical cancer screening (PARACAO)
Ye et al. [31]	2025	Nigeria	Type 2 hybrid interrupted time series	21,922 patients; 60 PHCs	Hypertension patients at 60 PHCs	WHO HEARTS package — Hypertension Treatment in Nigeria Program
Hennein et al. [36]	2021	Uganda	Qualitative (interviews + observation)	9 staff; TB clinics	TB clinic staff implementing integrated DM screening	Sustainability of DM screening integrated into TB clinics
Okpetu et al. [50]	2018	Nigeria	Descriptive mixed methods	Stakeholders + observed PHCs in FCT	Community representatives, HCWs, programme managers, FCT	Implementation of NCD component of National Minimum Health Care Package
Suglo et al. [30]	2024	Ghana	Two-phase qualitative + feasibility RCT	15 KIIs + 50 dyads (100 participants)	Adults with diabetes-related foot ulcer risk and family caregivers	Family-orientated diabetic foot-care intervention
Sansbury et al. [51]	2023	Malawi	Qualitative (IDIs)	39 participants; 10 NCD clinics	DMOs, NCD coordinators, providers in NCD clinics	Engaged leadership for integrated depression-NCD care
Mutabazi et al. [52]	2021	South Africa	Convergent mixed methods	30 interviewees + provincial PMTCT data	HIV/PMTCT experts, HCWs, women with HIV + GDM, Western Cape	Integration of GDM and T2DM care into PMTCT services
Kathree et al. [53]	2023	South Africa	Comparative group cohort	Adult PHC patients across 10 facilities	Adult PHC patients with chronic conditions	Task-shared collaborative care for depression in chronic disease services
David et al. [54]	2022	South Africa	Mixed methods	461 patients + 83 interviewees	T2DM patients at urban primary care facility, Cape Town	Community-based home delivery of medication during COVID-19
Katende et al. [55]	2023	Uganda	Cross-sectional comparative	222 HCWs + 420 patients; 22 facilities	HCWs and NCD patients at 22 PHC facilities	Medium-to-long-term sustainability of NCD service-readiness intervention
Songiso et al. [56]	2020	Zambia	Descriptive operational study	1,790 women	Symptomatic women at district-level breast care clinic, Lusaka	Integrated breast specialty care at PHC level
Lebina et al. [57]	2020	South Africa	Cross-sectional survey	90 staff; 6 PHC clinics	PHC staff in two health districts	Organisational culture and ICDM implementation fidelity
Lloyd-Sherlock et al. [38]	2018	South Africa	Pilot/feasibility study	20 participants ≥60 yrs	Older people in two villages, Agincourt, Mpumalanga	Pension-based engagement for hypertension management with low-sodium salt
Kushitor et al. [58]	2024	Ghana	Exploratory qualitative	12 HCWs + 26 clients; 3 hospitals	HCWs and clients of Wellness Clinics in deprived region	WHO-PEN Wellness Clinics for NCDs
Mahomed & Asmall [59]	2017	South Africa	Cross-sectional descriptive survey	264 nurses; 42 facilities	Professional nurses at ICDM-implementing PHC facilities	Professional nurses’ experiences with ICDM model
Malan et al. [60]	2015	South Africa	Qualitative	12 IDIs (nurses, doctors)	Primary care providers in South Africa	Brief behaviour-change counselling on NCD risk factors
Moyo et al. [61]	2023	Uganda	Qualitative programme evaluation	Field-notes-based	Patients and providers in HIV/DM/HTN integration sites	Scale-up of integrated HIV-NCD chronic care
Mash et al. [62]	2007	South Africa	Operational research study	400 patients with diabetes; 3 CHCs	Diabetes patients at 3 community health centres	Mobile fundal camera for diabetic retinopathy screening in PHC
Frieden et al. [63]	2020	Zimbabwe	Descriptive programme evaluation	3,094 patients; 9 PHCs + 2 hospitals	DM and HTN patients in rural Chipinge district	Nurse-led DM/HTN model in high-HIV-prevalence rural setting
Mundagowa et al. [64]	2024	Zimbabwe	Exploratory qualitative	38 (25 CHWs, 10 nurses, 3 admins)	CHWs, nurses, administrators at 5 PHCs, Mutare City	CHW-delivered home management of hypertension (HoMHyper)
Brooke-Sumner et al. [65]	2022	South Africa	Qualitative	Health managers (FGDs + IDIs)	Health managers, urban and rural PHC, Western Cape	Strategies for sustaining task-shared psychological intervention (Project MIND)
Badacho & Mahomed [66]	2023	Ethiopia	Cross-sectional (NHS-SM)	HIV/NCD team members; 5 PHCs	PHC HIV and NCD team members, southern Ethiopia	Sustainability of integrated HTN/DM with HIV care
Mahomed et al. [67]	2016	South Africa	Cross-sectional (NHS-SM)	37 PHC clinics; 3 districts	Staff at 37 ICDM-implementing PHC clinics	Sustainability of the ICDM model
Some et al. [68]	2016	Kenya	Descriptive retrospective review	725 nurse consultations; 616 patients	Stable NCD patients at 2 integrated PHCs, Kibera	Task-shifting NCD management to nurses
Labhardt et al. [69]	2010	Cameroon	Before–after programme evaluation	796 patients; 54 facilities	Patients with HTN/T2DM at non-physician clinician facilities, 8 rural districts	Task-shifting to non-physician clinicians
Godongwana et al. [70]	2021	South Africa	Phenomenological qualitative	24 HCPs and patients	PHC facilities implementing ICDM in two provinces	ICDM challenges in HIV/HTN/DM comorbidity
Ajisegiri et al. [12]	2022	Nigeria	Mixed methods case study	25 IDIs + 13 FGDs (107) + 13 facilities	HCWs, communities, PHC staff in 13 facilities, 4 states	Organisation of PHC delivery for NCDs
Oladele et al. [71]	2023	Nigeria	Pre-post training + qualitative	60 nurses and CHOs	Nurses and CHOs, Lagos State PHC Board	Task-strengthening for HTN management integrated into HIV care
Gausi et al. [72]	2021	South Africa	Pilot implementation evaluation (cohort)	247 multimorbid adults	Adults living with HIV and HTN/DM at 2 PHCs, Cape Town	Integrated ART-NCD care clubs
Ngassa Piotie et al. [73]	2022	South Africa	Single-group feasibility study	T2DM patients on max oral therapy	T2DM patients and PHC HCPs, City of Tshwane	Nurse-driven home-based telehealth for insulin therapy (TIP)
Kamvura et al. [35]	2021	Zimbabwe	ToC workshop/ participatory	18 stakeholders	Stakeholders in NCD and mental health care, Zimbabwe	Theory of Change for integrated depression-DM-HTN intervention (Friendship Bench)

### Quality assessment

For each study, an overall confidence level of high, moderate, or low was assigned. This reflects the completeness of reporting and methodological rigour rather than a numerical score, consistent with MMAT recommendations. Qualitative studies in the included evidence base demonstrated relatively high methodological coherence, with most meeting MMAT criteria for the appropriateness of the qualitative approach, the relevance of data collection methods, the derivation of findings from the data, the substantiation of interpretations, and the coherence between data sources and analysis. The pragmatic cluster RCT [29], the type 2 hybrid interrupted time series trial [31], and several large prospective and retrospective cohort studies [39,55,69] satisfied design-specific MMAT criteria for randomisation, completeness of outcome data, and management of confounders. Quantitative descriptive studies, which formed a substantial part of the evidence base, generally met criteria for sampling strategy and appropriate measurement, although several were limited in their handling of non-response or in the description of measurement validation. Mixed-methods studies showed variation in the integration of qualitative and quantitative strands, with the most rigorous studies clearly justifying their convergent or sequential design and presenting integrated findings. The two interventional studies described randomisation procedures but, as is typical for behavioural and health-systems interventions, provided limited detail on allocation concealment and blinding; both received moderate-to-high confidence ratings overall. Detailed item-level quality ratings are presented in S3 Table.

### Thematic synthesis

The synthesis was structured deductively around the four review questions and is therefore presented as four corresponding themes; within each theme, sub-themes were identified inductively from the included studies. First, six clusters of implementation strategies were identified, with task-shifting/task-sharing and integrated chronic care models the most prominent. Second, barriers and facilitators were predominantly health-system-level, with workforce, supply chain, leadership, policy operationalisation, and digital infrastructure all decisive. Third, implementation strategies were consistently associated with improvements in service delivery and provider knowledge, and with improvements in selected clinical outcomes. Fourth, scale-up and sustainability depended on system-level investment, embedded leadership, integration with the existing HIV chronic care platform, and iterative, context-adapted design (S5 Table).

#### Implementation strategies used to integrate NCD care into PHC.

The included studies described six broad clusters of implementation strategies, often used in combination rather than in isolation. Task-shifting and task-sharing were the single most frequently described strategies. Multiple studies described the delegation of stable NCD care to nurses, community health officers, and non-physician clinicians, with operational evaluations of task-shifting in Cameroon [69], Kenya [68], South Africa [29,53,65,67], Nigeria [71], and Zimbabwe [63] reporting feasibility and acceptable adherence to clinical protocols. Integrated chronic care models constituted a second prominent strategy, with the South African Integrated Chronic Disease Management (ICDM) model the most extensively studied [40,57,59,67,70], complemented by the Integrated Chronic Care Clinic (IC3) model in rural Malawi [39], integrated HIV-NCD club models in Cape Town [72], and integrated chronic care platforms in Uganda [61] and southern Ethiopia [66]. The ICDM model is a South African model of care that reorganises primary-care delivery to manage chronic communicable (principally HIV) and non-communicable conditions together within a single facility-based system; its core components include facility reorganisation, clinical support and supervision, assisted patient self-management, and strengthened supply and information systems. Workforce capacity-building, training, and educational outreach were widely deployed: Mozambique implemented a cascade Training-of-Trainers approach for cardiovascular risk [46]; Uganda implemented a national capacity-building programme for cancer prevention, reaching 488 PHC workers across 118 districts [44]; South Africa evaluated the PC101 educational outreach programme with an integrated clinical management tool in a pragmatic cluster RCT [29]; Nigeria implemented hybrid virtual-onsite training for hypertension management integrated into HIV care [71]; and South Africa evaluated brief behaviour-change counselling training [60].

Digital health and decision-support tools formed a fourth cluster of strategies. Smartphone-based mentorship for cervical cancer screening using visual inspection with acetic acid (VIA) was implemented in Eswatini, with weekly remote expert review of cervical images captured by nurses producing high agreement rates and reduced false positives [47]. A mobile fundal camera was deployed in three South African community health centres for diabetic retinopathy screening, with operational data demonstrating that a single technician could photograph approximately 10,000 patients per year [62]. A nurse-driven home-based telehealth intervention improved insulin therapy and glycaemic control among South African patients with type 2 diabetes [73], and HPV-based cervical cancer screening with electronic decision-support was implemented in Burkina Faso [32]. Community- and group-based delivery models constituted a fifth cluster, including hypertension clubs in South Africa [49], group empowerment for type 2 diabetes [48], pension-delivery-point engagement for older adults with hypertension [38], CHW home-management of hypertension in Zimbabwe [64], CHW home delivery of medication during the COVID-19 pandemic [54], a microfinance-group medical visit model in rural Kenya developed using human-centred design [34], and theory-of-change-derived integrated NCD-mental health interventions building on the Friendship Bench in Zimbabwe [35]. The sixth cluster concerned the implementation of guidelines, protocols, and quality-improvement packages, including the Ethiopian Primary Healthcare Clinical Guidelines [43], the Western Cape Adult Chronic Disease Management Policy [42], the WHO HEARTS package as deployed in the Hypertension Treatment in Nigeria Program [31], NCD integration into emergency primary care in conflict-affected DRC [45], breast specialty services at PHC level in Zambia [56], and the NCD component of Nigeria’s National Minimum Health Care Package in the Federal Capital Territory [50].

A cross-cutting observation was that most studies described not single strategies but bundled, multi-component implementation packages. Task-shifting succeeded only where training, supervision, equipment, and protocols were also provided [63,68,69]; integrated chronic care models depended on workforce reconfiguration and clinical guideline implementation in addition to physical service co-location [39,59,69,72]; and digital tools were generally embedded in broader workforce capacity-building programmes [29,47,73]. This observation is consistent with implementation science evidence that bundled, context-adapted strategies are typically more effective than single-strategy interventions [24,26]. These clusters and their correspondence to the ERIC taxonomy are summarised in Table 2.

**Table 2:** Mapping of the six implementation‑strategy clusters to the Expert Recommendations for Implementing Change (ERIC) taxonomy.

Strategy cluster (this review)	Representative ERIC discrete strategies	ERIC category (Waltz et al. [22])	Illustrative examples from included studies
**1. Task-shifting and task-sharing**	Revise professional roles; Create new clinical teams; Provide clinical supervision; Conduct ongoing training	Support clinicians; Provide interactive assistance; Train and educate stakeholders	Delegation of stable hypertension/diabetes care to nurses and non-physician clinicians with supervision and simplified protocols [53,63,68,69,71]
**2. Integrated chronic care models**	Change service sites; Create new clinical teams; Revise professional roles; Change record systems; Promote adaptability	Change infrastructure; Support clinicians; Adapt and tailor to context	ICDM and IC3 models co-locating HIV and NCD services with shared registers and reorganised teams [39,40,59,66,72]
**3. Workforce capacity-building, training and educational outreach**	Conduct educational outreach visits; Conduct ongoing training; Develop and distribute educational materials; Use train-the-trainer strategies; Conduct educational meetings	Train and educate stakeholders	PC101 educational outreach [29]; cascade training-of-trainers [46]; national cancer-control capacity-building [44]; hybrid task-strengthening training [71]
**4. Digital health and decision-support tools**	Facilitate relay of clinical data to providers; Remind clinicians; Change record systems; Centralize/provide technical assistance	Support clinicians; Change infrastructure; Provide interactive assistance	Smartphone-based remote VIA review [47]; mobile fundal camera [62]; nurse-driven telehealth for insulin [73]; electronic decision support for HPV screening [32]
**5. Community- and group-based delivery models**	Involve patients/consumers and family members; Prepare patients to be active participants; Intervene to enhance uptake and adherence; Change service sites	Engage consumers; Change infrastructure	Group empowerment for type 2 diabetes [48]; family-oriented foot-care [30]; CHW home delivery/home management [54,64]; pension-point engagement [38]
**6. Guidelines, protocols and quality-improvement packages**	Develop a formal implementation blueprint; Develop and distribute educational materials; Audit and provide feedback; Develop and implement tools for quality monitoring; Conduct cyclical small tests of change; Tailor strategies	Use evaluative and iterative strategies; Train and educate stakeholders; Adapt and tailor to context	Ethiopian PHC Clinical Guidelines [43]; WHO HEARTS package [31]; NCD integration into emergency PHC [45]; National Minimum Health Care Package [50]

#### Barriers and facilitators influencing implementation.

Barriers and facilitators identified across the included studies were predominantly health-system-level. Barriers and facilitators were classified by ecological level (individual/provider, organisational/facility, and health-system) drawing on the levels articulated in the Consolidated Framework for Implementation Research (CFIR) [24] and related implementation-science perspectives [25]. Workforce shortages and competing demands emerged as the most consistent barrier, reported across studies in Ethiopia [43], Zimbabwe [37,64], South Africa [40,59,60,65,70], and Malawi [51]. Frontline providers described working in environments of high patient volume, multiple competing programmes, and insufficient time to deliver guideline-based NCD care [37,60]. Supply chain and infrastructure constraints, particularly stock-outs of essential medicines and reagents, were the second consistent barrier and the most decisive in several settings. In Ethiopia, the non-availability of medicines and laboratory reagents was identified as a key barrier to implementation of the Ethiopian PHC Clinical Guidelines [43]. In Uganda, a national supply-chain disruption for glucose test strips ended a previously functioning DM screening programme integrated within TB clinics, with cascading effects on clinic capacity and provider self-efficacy [36]. By contrast, the Hypertension Treatment in Nigeria Program demonstrated that establishing a drug revolving fund could maintain medication availability in 95% of participating PHCs [31], and free medication distribution stabilised availability in the rural Zimbabwe nurse-led model [63].

Leadership, organisational climate, and culture emerged as a third decisive set of factors, operating predominantly as facilitators rather than barriers. In Malawi, engaged, approachable leaders who were involved in daily operations and modelled supportive behaviour were identified as key drivers of integrated depression-NCD care [51]. Organisational culture, particularly the involvement, adaptability, and consistency dimensions of the Denison Organisational Culture framework, predicted ICDM implementation fidelity in South Africa [57]. Sustainability assessments using the NHS Sustainability Model in South Africa and southern Ethiopia identified clinical leadership engagement as a major determinant of long-term ICDM and integrated HIV-NCD-care performance [66,67]. Project MIND in South Africa identified the relational work that managers undertake with staff, promoting support and reducing resistance, as a critical lever for sustained implementation of task-shared psychological interventions [65].

Policy alignment and guideline operationalisation formed a fourth set of factors. A persistent gap between policy and practice was identified in South Africa [42], Nigeria [12,50], Ethiopia [43], and Ghana [58]. Common contributors were poor knowledge of policy among frontline clinicians [42], unclear delineation of roles [12,71], and limited operational tools for translating policy into routine practice [43,50]. Patient and community factors emerged as a fifth set of moderators of acceptability and uptake. Health literacy [30,41], stigma and disease denial [41], financial hardship [59], and acceptability of the materials and modes of delivery [30,38,41] all shaped uptake. Iteratively designed, culturally adapted, and family-oriented interventions in Ghana [30] and Kenya [34] illustrated how patient-level barriers could be partially addressed through implementation design. Digital infrastructure and connectivity were a sixth, increasingly important moderator. Digital interventions extended reach but were dependent on connectivity, device availability, and digital literacy among providers [32,47,63,73]; failures of these foundations limited the scalability of otherwise effective digital tools.

#### Effects on service delivery and patient outcomes.

Implementation strategies were associated with varied but largely positive effects across implementation, service delivery, and patient outcome domains. In service delivery and access terms, integrated chronic care models substantially expanded NCD service availability and uptake. The IC3 model in Malawi enrolled 6,233 patients across 14 PHC facilities and achieved 72% one-year retention for patients with NCDs, with statistically significant clinical improvements for hypertension, diabetes, asthma, and epilepsy [39]. Integration of NCD management into emergency primary care in the conflict-affected Beni Region of DRC was feasible at programme and management costs of approximately USD 115 per person per treatment course, with consultations for hypertension and diabetes increasing significantly before the conflict-related disruption [45]. Task-shifting expanded access in Cameroon [69], Kenya [68], and rural Zimbabwe [63], and breast specialty services at the PHC level in Zambia identified primary breast cancer in 64% of women referred for histological evaluation, with surgery for early-stage cancers performed within two weeks of pathological diagnosis [56].

Provider knowledge and competence improved consistently following training-based strategies. The Mozambican Training-of-Trainers cascade improved mean pre- and post-test scores from 53% to 90% in the first workshop and from 59% to 78% and 58% to 74% in subsequent workshops, with new local trainers reporting confidence increases on a 5-point Likert scale from 3.0 to 4.8 [46]. Capacity-building in Uganda trained 488 PHC workers across 118 districts and led to 75% of districts implementing at least one agreed action at follow-up [44]. The Nigerian hybrid task-strengthening training improved hypertension management knowledge from a mean of 59.0% pre-test to 67.6% post-test [71]. The PC101 educational outreach programme in South Africa was feasible and safe, and was adopted nationally despite not demonstrating superiority over PALSA PLUS (Practical Approach to Lung Health and HIV/AIDS in South Africa) for treatment intensification or depression case detection [29].

Clinical outcomes for hypertension improved within integrated and task-shifted models. The South African hypertension club reduced the proportion of patients with blood pressure above 140/90 mmHg from 48% at baseline to 33% at six months [49]. The Hypertension Treatment in Nigeria Program increased fixed-dose combination prescribing from 16.3% to 65.2% and improved 6-month patient retention from 59.9% to 63.1% [31]. The system dynamics modelling study in Nigeria identified health-care worker (HCW) training as the most effective single strategy for retention (29.7% at 12 months), with integrated intervention packages producing the greatest improvements (72.4% at 12 months) [33]. The Cameroon non-physician clinician programme reduced systolic blood pressure by 22.8 mmHg and fasting plasma glucose by 3.4 mmol/L at programme assessment [69]. Effects on diabetes control were generally less consistent than effects on blood pressure: glycaemic control was suboptimal in both arms of the South African community-based home-delivery study despite significant improvements during the COVID-19 pandemic [54], and the integrated care clubs in Cape Town achieved optimal glycaemic control in only around 50% of patients in the year following enrolment [72]. The Tshwane Insulin Project feasibility study reduced HbA1c by 2.2% (95% CI 1.6–2.8%) using a nurse-driven, home-based telehealth approach [73].

Patient experience and acceptability outcomes were generally favourable. Patient education was rated highly acceptable in iHEART-SA, with simple language, knowledge empowerment, and perceived health improvements as key facilitators [41]. The family-oriented diabetic foot-care intervention in Ghana met all feasibility criteria and demonstrated efficacy signals favouring the intervention group on improved foot-care behaviour, foot self-care efficacy, diabetes knowledge, and reduced diabetes distress at 12 weeks post-randomisation [30]. The pension-delivery-point intervention in rural South Africa retained 19 of 20 participants over three months and achieved high acceptability of low-sodium salt with repeat usage [38]. Group empowerment for type 2 diabetes at Grassy Park Community Day Centre achieved high overall attendance (65% of invited patients attended at least one session) but lower full-attendance rates (29% attended all four sessions) [48]. Implementation outcomes, including fidelity, adoption, and short-term sustainability, were generally achievable, but longer-term sustainability was vulnerable to interruptions: the Ugandan tuberculosis–diabetes (TB–DM) integrated screening programme ceased entirely after national supply-chain disruption for glucose test strips [36]; medium-to-long-term Ugandan NCD service-readiness intervention performance declined modestly four years post-trial despite preserved quality of hypertension and diabetes care for 88% of patients [55]; and ICDM sustainability in South Africa varied widely across districts, with 11% of facilities scoring below the 55-point threshold for sustainability on the NHS Sustainability Model [67]. S6 Table maps the six implementation strategy clusters to the implementation, service delivery, and patient outcomes reported across the included studies, illustrating how individual strategies contributed to observed effects across these domains.

#### Implications for scale-up, sustainability, and health system strengthening.

Five implications for scale-up and sustainability emerged from the synthesis. First, leveraging the HIV chronic care platform was identified as one of the most feasible and efficient routes to scaling up integrated NCD care. The IC3 model built on Malawi’s HIV infrastructure facilitated rapid decentralisation of NCD services in rural settings [39]; the integrated care clubs in Cape Town maintained HIV viral suppression while introducing hypertension and diabetes management for HIV-positive multimorbid adults [72]; the integration of gestational and type 2 diabetes care into prevention of mother-to-child transmission (PMTCT) services in the Western Cape provided a feasible template for chronic disease prevention extending from maternal-child platforms [52]; and the southern Ethiopian and Ugandan integrated platforms demonstrated similar feasibility in lower-resource contexts [61,66,71]. Second, embedded leadership and continuous mentorship were identified across multiple settings as preconditions for sustained implementation [30,35,57,63,65,67], suggesting that scale-up strategies that focus only on the technical content of interventions, without parallel investment in leadership development and organisational culture change, are unlikely to achieve durability.

Third, financing and supply-chain investment were decisive. Drug revolving funds and pre-positioned supplies, where established, stabilised availability and protected gains [31,63]. Episodic and donor-dependent financing, by contrast, placed sustainability at risk [36] and contributed to the documented attrition of providers, equipment, and reagents in several settings [43,64,67]. Fourth, iterative, context-adapted scale-up was associated with both feasibility and acceptability. Human-centred design in rural Western Kenya produced a microfinance-group medical visit model that addressed medication availability, financial resources, peer support, and caregiver burden in a single integrated package [34]. The theory-of-change approach used by the Friendship Bench in Zimbabwe identified expert clients with lived experience as central to a patient-centred design and identified WhatsApp as an alternative digital platform during the COVID-19 disruption [35]. The convergent mixed-methods evaluation of Group Empowerment and Training (GREAT) in Cape Town identified 30 determinants of successful implementation through prospective programme theory work [48]. Fifth, integration with health system strengthening priorities (including workforce planning, governance, financing, supply chain management, and information systems) was repeatedly identified as a precondition for the durable scale-up of integrated NCD care [12,43,50,55,58,60].

Across these five themes, the synthesis shows that integration of NCD care into PHC in SSA is fundamentally a health system strengthening intervention rather than a discrete clinical innovation. The strategies that succeeded most consistently across diverse contexts were those that combined task-shifting or integrated chronic care with capacity-building, supply-chain investment, leadership development, and iterative adaptation. The strategies that failed most consistently were those that relied on a single mechanism, such as training without supply-chain support or digital tools without infrastructure, or that depended on episodic external funding without integration into national PHC delivery and financing arrangements. The strength of these conclusions is reflected in the certainty-of-evidence assessments. An overview of the certainty of the evidence for each theme is presented in Table 3, with detailed per-finding assessments provided in the S4 Table.

**Table 3:** Summary of findings and certainty of the evidence, by theme.

Theme (key conclusion)	Contributing studies (n; design)	Methodological limitations	Consistency of evidence	Overall certainty
Theme 1. Six clusters of implementation strategies are used to integrate NCD care into PHC – task‑shifting/ task‑sharing; integrated chronic care models; workforce capacity‑building, training and educational outreach; digital health and decision‑support tools; community‑ and group‑based delivery; and guidelines, protocols and quality‑improvement packages – most often deployed as bundled, multi‑component packages. Task‑shifting/ task‑sharing and integrated chronic care models were the most prominent.	All 46 included studies (each categorised into ≥1 strategy cluster). Designs: 1 pragmatic cluster RCT, 1 feasibility RCT, 2 hybrid effectiveness/ interrupted time series, plus cohort, before–after, cross‑sectional, qualitative and mixed‑methods studies.	Moderate. Non‑randomised and descriptive designs predominate; controlled designs are a minority.	High for the characterisation of the six clusters; strategies consistently described as bundled and multi‑component.	Moderate Range: Low–Moderate to Moderate–High across the six clusters; highest for task‑shifting/ task‑sharing.
Theme 2. Barriers and facilitators are predominantly health‑system level. The most consistent barriers were workforce shortages and competing demands, medication and supply‑chain interruptions, and a persistent policy–practice gap; the most consistent facilitators were engaged frontline leadership, a supportive organisational climate, community partnerships, and drug revolving funds.	Predominantly qualitative and mixed‑methods studies reporting implementation barriers and facilitators, supported by operational data (supply‑chain records, sustainability instruments); converging across ≥10 countries.	Minor. Predominantly high‑quality qualitative studies on MMAT appraisal.	High. Findings converge across countries and multiple study designs.	High Range: Moderate to High; the strongest component of the evidence base.
Theme 3. Implementation strategies were associated with improved service delivery and access, provider knowledge and competence, and patient acceptability, and with improved clinical control for hypertension. Effects on glycaemic control, retention in care, and long‑term sustainability were less consistent.	Predominantly observational (cohort, before–after, descriptive) with 1 cluster RCT and 1 hybrid interrupted time series; single‑arm pre–post designs for provider‑knowledge outcomes.	Moderate to serious for selected outcomes. Many single‑arm/ uncontrolled designs; few randomised comparisons.	Consistent for BP control, provider knowledge and acceptability; inconsistent for glycaemic control, retention and sustainability.	Moderate/ Low Moderate for hypertension control, service delivery and provider knowledge; Low for glycaemic control, retention and long‑term sustainability.
Theme 4. Durable scale‑up and sustainability depend on system‑level investment (workforce, supply chains, information systems), embedded leadership and continuous mentorship, predictable financing such as drug revolving funds, integration with the existing HIV chronic care platform, and iterative, context‑adapted design.	Cohort, operational, qualitative and mixed‑methods studies, including a natural experiment of programme interruption and sustainability‑instrument (NHS‑SM) studies; multi‑country.	Moderate. Operational and cross‑sectional designs predominate.	Consistent across multi‑country operational and qualitative evidence.	Moderate Range: Moderate to Moderate–High.

## Discussion

Our systematic review synthesised evidence on implementation strategies for integrating NCD care into PHC in SSA. Across 46 studies from 14 countries, six clusters of strategies were identified: task-shifting and task-sharing, integrated chronic care models, workforce capacity-building, digital health and decision-support tools, community- and group-based delivery, and the implementation of guidelines, protocols, and quality-improvement packages. These strategies were rarely deployed in isolation. Successful examples in the included evidence were multi-component packages in which task-shifting was supported by training, supervision, equipment such as blood-pressure monitors, glucometers, point-of-care HbA1c devices, and, for screening programmes, mobile fundal and cervical-imaging cameras [63,68,69]. Integrated chronic care models were supported by workforce reconfiguration and guideline implementation. Digital tools were not deployed in isolation but were paired with provider training and supervision, for example, remote expert mentorship accompanying smartphone-based cervical screening, and nurse training accompanying telehealth-supported insulin initiation [29,47,73]. This pattern is consistent with implementation science evidence from other regions [24,26].

Task-shifting emerged as the most frequently described strategy. Across diverse settings, including Kenya [68], Cameroon [69], South Africa [29,53,59,65], Nigeria [71], and Zimbabwe [63], the delegation of stable NCD care to nurses, community health officers, and non-physician clinicians was feasible and was associated with clinically meaningful improvements in blood pressure control. Cluster-randomised evidence from South Africa demonstrated that nurse-led management with integrated clinical decision-support was feasible and safe, although it did not produce superior outcomes to the active comparator on treatment intensification [29]. Nurse-led models in rural Zimbabwe achieved registration of 3,094 patients with hypertension and diabetes across nine PHC facilities and two hospitals with structured intensive mentoring and free medication [63]. These findings are consistent with broader systematic-review evidence that task-shifting for NCDs in LMICs can produce clinically meaningful improvements in selected outcomes [74,75], and contribute new SSA-specific operational evidence on the conditions under which task-shifting succeeds.

The evidence on integrated chronic care models is broadly positive but reveals important caveats. Where the South African ICDM model and the Malawian IC3 model were implemented with adequate workforce, supply-chain, and leadership support, they expanded NCD service availability and produced clinically meaningful improvements [39,40,59]. However, fidelity to the ICDM model was sensitive to organisational culture [57], staff workload [40,59], policy operationalisation [42], and the spatial co-location of TB, HIV, and NCD services in shared facility space [40]. The challenges of HIV-NCD multimorbidity in ICDM facilities; pill burden, non-disclosure, financial burden, and unclear chronic-comorbidity guidelines, were identified as persistent gaps [70]. These findings indicate that integrated chronic care is not a single, replicable intervention but a system-level transformation whose success depends on the simultaneous alignment of multiple system components.

The barriers and facilitators identified across the synthesis are consistent with established implementation science frameworks and also reveal specific patterns in SSA. Workforce shortages and supply-chain interruptions emerged as the most consistent and decisive barriers, which accords with broader evidence on health-system constraints in the region [76,77]. Engaged frontline leadership and supportive organisational culture emerged as the most consistent and powerful facilitators [51,57,65,67]. This echoes implementation science evidence on the centrality of leadership and the inner setting in shaping uptake [24]. The gap between national policy and frontline practice observed in South Africa [42], Nigeria [12,50], Ethiopia [43], and Ghana [58] points to a recurring challenge in translating policy commitments into operational change at the PHC level. The increasing prominence of digital tools, with strong demonstrations of feasibility for cervical cancer screening [32,47], diabetic retinopathy screening [62], and insulin therapy support [73], must be balanced against the dependence of such interventions on connectivity, device availability, and digital literacy.

Effects on clinical outcomes were positive overall but uneven across conditions. Hypertension control improved consistently across multiple settings and study designs [31,33,49,63,69], with effect sizes that were sometimes substantial. Diabetes control improved in some integrated and task-shifted models [39,54,69,73] but was less consistent overall [54,72]. Patient experience and acceptability outcomes were consistently positive where measured [30,38,41,48,54], with patient-centred and culturally adapted designs producing the strongest acceptance. Sustainability outcomes were the most challenging area: short-term sustainability was generally achievable, but longer-term sustainability was vulnerable to supply-chain disruption [36], reduced supervision [55], and weak progress monitoring [66,67]. This pattern echoes broader concerns in implementation science about the fragility of programmes that rely on episodic external support [25].

Reading across the three preceding themes reveals coherent strategy–barrier–outcome relationships. Where supply-chain barriers were directly addressed through drug revolving funds or free medication, task-shifted and integrated models sustained medication availability and blood-pressure control [31,63], whereas comparable models without such measures faltered when supplies were interrupted [36]. Where workforce and workload barriers were met with training, supervision, and leadership engagement, integrated chronic care models achieved fidelity and retention [39,57], while their absence undermined both [59,70]. That outcomes tracked whether the corresponding system-level barrier was addressed reinforces the review’s central finding: integration succeeds as a health-system intervention rather than a discrete clinical one, and sustainability depends on designing strategies around the barriers most binding in each setting.

Several caveats deserve emphasis. The geographic distribution of the included studies is uneven, with South Africa accounting for over 40% of the evidence base. This concentration partly reflects the strength of implementation research capacity in South Africa, as well as publication patterns and language constraints. The implications for generalisability are important. South Africa operates with a stronger health system architecture, a more developed primary care policy framework, and greater research capacity than many other SSA countries. The findings from South African studies, particularly on ICDM fidelity, sustainability, and task-sharing, may not translate directly to settings with weaker health system foundations, such as those in Central or parts of West and East Africa, which are underrepresented in this review. This imbalance may also introduce optimism bias, as the review disproportionately reflects implementation experiences from comparatively well-resourced settings where successful integration is more likely to be achieved and sustained. Accordingly, the feasibility and sustainability of these approaches in fragile, conflict-affected, or resource-constrained settings should be interpreted cautiously. Expanding implementation research in underrepresented countries should therefore be a priority to strengthen the regional evidence base and support contextually appropriate adaptation of integration strategies.

From a health equity perspective, integration strategies were frequently deployed among populations that are otherwise underserved. Much of the evidence came from rural and remote settings, where nurse- and community-health-worker-led models extended NCD care to populations distant from physician-staffed facilities [63,64,68,69]. Several strategies targeted specific vulnerable groups: pension-linked engagement and low-sodium-salt provision for older adults [38]; cervical and breast cancer screening for women [32,47,56]; family-oriented and group-based models addressing caregiver burden and financial hardship [30,54]; and integrated HIV-NCD platforms for people living with HIV and multimorbidity [39,61,66,67,72]. Integration was also feasible in a fragile, conflict-affected setting, where NCD care was embedded within emergency primary care in the Democratic Republic of the Congo [45]. At the same time, the evidence exposes equity risks: the strategies most dependent on digital connectivity, device availability, and reliable supply chains are precisely those most likely to falter in the poorest and most remote settings, potentially widening rather than narrowing gaps unless the underlying infrastructure is addressed.

From a policy perspective, the findings have direct implications for progress towards SDG 3.4 and 3.8, which aim to reduce premature NCD mortality by one-third by 2030, and to achieve universal health coverage with access to quality essential healthcare services, respectively [13]. The synthesis demonstrates that integrated NCD care delivered through PHC is feasible across diverse SSA contexts and can produce meaningful improvements in service availability, provider knowledge, and selected clinical outcomes. Achieving these goals will require investment in workforce, supply chains, information systems, and leadership development, alongside strategic alignment with the existing HIV chronic care platform. National NCD strategies in SSA should explicitly position implementation strategies not merely as clinical interventions but as a core component of integration plans, with measurable indicators of fidelity, sustainability, and equity.

### Limitations

This review has several limitations. Only studies published in English were included, which may have excluded relevant evidence from Francophone and Lusophone countries in SSA. The review was also restricted to PubMed/MEDLINE, AJOL, and Google Scholar. We did not search Embase, Scopus, Web of Science, CINAHL, Global Health, or the Cochrane Library, and it is possible that studies indexed only in those databases were missed. Moreover, the included studies were unevenly distributed across the region, with most evidence originating from South Africa, Nigeria, Zimbabwe, Uganda, Malawi, Ethiopia, and Kenya, while countries in Central and parts of West Africa were underrepresented.

Furthermore, differences in implementation strategies, reporting approaches, and outcome measures across studies limited direct comparison and made meta-analysis inappropriate. Implementation outcomes, particularly sustainability, were also measured inconsistently across the studies reviewed. Although the MMAT was used to assess methodological quality, it does not specifically evaluate risk of bias in causal relationships, especially within cross-sectional and descriptive studies, which formed a large proportion of the included evidence.

## Conclusion

Our review shows that implementation strategies for integrating NCD care into PHC in SSA fall into six broad clusters: task-shifting and task-sharing, integrated chronic care models, workforce capacity-building, digital health and decision-support tools, community- and group-based delivery, and implementation of guidelines, protocols, and quality-improvement packages. These strategies appear most effective when implemented as bundled and context-adapted interventions rather than as single approaches.

The main barriers to implementation were workforce shortages, supply-chain interruptions, and weak operationalisation of guidelines. Key facilitators included engaged leadership, a supportive organisational climate, integration with existing HIV chronic care platforms, and continuous adaptation during implementation. Integration was associated with improvements in service delivery and provider competence. It was also associated with improvements in some clinical outcomes, particularly hypertension control. However, findings on diabetes control, patient retention, and long-term sustainability were less consistent. Successful scale-up will require sustained investment in the health workforce, supply chains, information systems, and leadership development. It will also require iterative and context-sensitive programme design that aligns with national PHC strategies. These efforts are important for achieving Sustainable Development Goals 3.4 and 3.8 and for strengthening resilient, integrated PHC systems in SSA. Future research should focus on economic evaluations of integration strategies, long-term evidence on sustainability, and equity-disaggregated outcomes. Such evidence will help guide the design and scale-up of feasible and contextually appropriate integrated NCD care models across the region.

## Supporting information

S1 Checklist PRISMA 2020 checklist. Adapted from Page MJ, McKenzie JE, Bossuyt PM, et al. The PRISMA 2020 statement: an updated guideline for reporting systematic reviews. BMJ 2021;372:n71. Licensed under CC BY 4.0.
(DOCX)

S1 Text Literature search strategy. (DOCX)

S1 Table Summary of eligibility criteria. (DOCX)

S2 Table Data extraction records. (DOCX)

S3 Table Quality appraisal of included studies (MMAT). (DOCX)

S4 Table Certainty of evidence. (DOCX)

S5 Table Thematic analysis result. (DOCX)

S6 Table Strategy outcomes matrix. (DOCX)

S7 Table Excluded studies. (DOCX)
