# Supplementary material for: Implementation strategies for integrating non-communicable disease care into primary healthcare settings in sub-Saharan Africa: A systematic review
Source: PLOS Glob Public Health. 2026 Sep 15;6(9):e0007062. doi: 10.1371/journal.pgph.0007062 (PMC13577421; doi:10.1371/journal.pgph.0007062)
Supplement: Supplementary file 2 [file pgph.0007062.s002.docx]

**Search strategy**

**PubMed/MEDLINE**

**Concept blocks:** (NCD terms) AND (PHC terms) AND (integration/implementation terms) AND (sub-Saharan Africa terms)

("Noncommunicable Diseases"[Mesh] OR noncommunicable[tiab] OR "non communicable"[tiab] OR "chronic disease"[tiab] OR "chronic illness"[tiab] OR "mental health"[tiab] OR "mental disorder"[tiab] OR depression[tiab] OR "anxiety disorder"[tiab] OR hypertension[tiab] OR "high blood pressure"[tiab] OR diabetes[tiab] OR "diabetes mellitus"[tiab] OR "cardiovascular disease"[tiab] OR stroke[tiab] OR cancer[tiab] OR neoplasm[tiab] OR "chronic respiratory disease"[tiab] OR COPD[tiab] OR asthma[tiab])

AND

("Primary Health Care"[Mesh] OR "primary health care"[tiab] OR "primary healthcare"[tiab] OR "primary care"[tiab] OR "community health service*"[tiab] OR "community health center*"[tiab] OR "family practice"[tiab] OR "general practice"[tiab] OR PHC[tiab])

AND

("Delivery of Health Care, Integrated"[Mesh] OR integrat*[tiab] OR "integrated care"[tiab] OR "service integration"[tiab] OR "integrated service*"[tiab] OR "care integration"[tiab])

AND

(implement*[tiab] OR "implementation strateg*"[tiab] OR "implementation outcome*"[tiab] OR "scale up"[tiab] OR "scale-up"[tiab] OR sustain*[tiab] OR "task shifting"[tiab] OR "task sharing"[tiab] OR decentrali*[tiab] OR "guideline implementation"[tiab] OR "quality improvement"[tiab] OR mHealth[tiab] OR "digital health"[tiab] OR telemedicine[tiab])

AND

("Africa South of the Sahara"[Mesh] OR "sub-Saharan Africa"[tiab] OR "sub Saharan Africa"[tiab] OR subsaharan[tiab] OR SSA[tiab] OR Angola[tiab] OR Benin[tiab] OR Botswana[tiab] OR "Burkina Faso"[tiab] OR Burundi[tiab] OR "Cabo Verde"[tiab] OR "Cape Verde"[tiab] OR Cameroon[tiab] OR "Central African Republic"[tiab] OR Chad[tiab] OR Comoros[tiab] OR Congo[tiab] OR "Democratic Republic of the Congo"[tiab] OR DRC[tiab] OR "Republic of the Congo"[tiab] OR "Cote d'Ivoire"[tiab] OR "Ivory Coast"[tiab] OR Djibouti[tiab] OR "Equatorial Guinea"[tiab] OR Eritrea[tiab] OR Eswatini[tiab] OR Swaziland[tiab] OR Ethiopia[tiab] OR Gabon[tiab] OR Gambia[tiab] OR Ghana[tiab] OR Guinea[tiab] OR "Guinea-Bissau"[tiab] OR Kenya[tiab] OR Lesotho[tiab] OR Liberia[tiab] OR Madagascar[tiab] OR Malawi[tiab] OR Mali[tiab] OR Mauritania[tiab] OR Mauritius[tiab] OR Mozambique[tiab] OR Namibia[tiab] OR Niger[tiab] OR Nigeria[tiab] OR Rwanda[tiab] OR "Sao Tome and Principe"[tiab] OR Senegal[tiab] OR Seychelles[tiab] OR "Sierra Leone"[tiab] OR Somalia[tiab] OR "South Africa"[tiab] OR "South Sudan"[tiab] OR Tanzania[tiab] OR Togo[tiab] OR Uganda[tiab] OR Zambia[tiab] OR Zimbabwe[tiab])

AND

("1990/01/01"[Date - Publication] : "2025/12/31"[Date - Publication])

**Google Scholar**

("non-communicable disease" OR "noncommunicable disease" OR NCD OR hypertension OR diabetes OR "cardiovascular disease" OR cancer OR "chronic respiratory disease" OR asthma OR COPD OR “mental health” OR mental disorder OR depression OR anxiety disorder)

AND

("primary healthcare" OR "primary health care" OR "primary care" OR "community health services" OR "family practice")

AND

("integrated care" OR integration OR "service integration" OR "implementation strategy" OR "implementation strategies" OR "task shifting" OR "task sharing" OR decentralisation OR decentralisation OR "integrated chronic care" OR "clinical guideline*" OR "quality improvement" OR mHealth OR telemedicine OR "digital health")

AND

("sub-Saharan Africa" OR "Sub Saharan Africa" OR SSA OR Angola OR Benin OR Botswana OR "Burkina Faso" OR Burundi OR Cameroon OR "Cape Verde" OR "Central African Republic" OR Chad OR Comoros OR Congo OR "Democratic Republic of Congo" OR Djibouti OR Eritrea OR Eswatini OR Ethiopia OR Gabon OR Gambia OR Ghana OR Guinea OR "Guinea-Bissau" OR Kenya OR Lesotho OR Liberia OR Madagascar OR Malawi OR Mali OR Mauritania OR Mauritius OR Mozambique OR Namibia OR Niger OR Nigeria OR Rwanda OR Senegal OR Seychelles OR "Sierra Leone" OR Somalia OR "South Africa" OR "South Sudan" OR Sudan OR Tanzania OR Togo OR Uganda OR Zambia OR Zimbabwe)

**African Journal online**

| S/N | Strategy | **Results** |
| --- | --- | --- |
| 1. | hypertension AND primary healthcare | 53 |
| 2. | noncommunicable disease AND primary healthcare | 297 |
| 3. | diabetes AND primary healthcare | 77 |
| 4. | mental health AND primary healthcare | 1,013 |
| 5. | "task shifting AND noncommunicable | 2,618 |
| 6. | noncommunicable disease AND digital health | 125 |
| 7. | chronic disease AND primary healthcare | 580 |
|  |  | **4,763** |
