# Supplementary material for: Implementation strategies for integrating non-communicable disease care into primary healthcare settings in sub-Saharan Africa: A systematic review
Source: PLOS Glob Public Health. 2026 Sep 15;6(9):e0007062. doi: 10.1371/journal.pgph.0007062 (PMC13577421; doi:10.1371/journal.pgph.0007062)
Supplement: Supplementary file 3 [file pgph.0007062.s003.docx]

**Summary of Eligibility criteria for study inclusion, structured by the Population–Exposure–Outcome (PEO) framework.**

| **Criterion (PEO element)** | **Inclusion criteria** | **Exclusion criteria** |
| --- | --- | --- |
| **Population** | Individuals receiving NCD‑related services (prevention, screening, diagnosis, treatment, follow‑up, or chronic care) at the PHC level; and/or health workers and PHC teams delivering NCD care (medical doctors, nurses, midwives, community health workers, physician assistants, and other non‑physician providers). | Populations receiving NCD care exclusively outside PHC; studies not involving NCD care recipients or PHC providers. |
| **Exposure (implementation strategy)** | One or more implementation strategies or delivery models aimed at integrating NCD care into PHC, including: task‑shifting / task‑sharing; decentralisation of services; integrated chronic care models; clinical guideline/protocol implementation; workforce‑strengthening interventions; referral‑pathway strengthening; digital health and decision‑support tools (mHealth, telemedicine); and community‑based PHC programmes targeting NCDs. | No implementation or integration strategy described. For example, studies reporting only disease burden, risk factors, or the clinical effectiveness of a treatment without an implementation or integration component. |
| **Outcomes** | The implementation strategy itself was the primary outcome (its description, actors, actions, setting, and duration of application).  The primary outcome of interest was the implementation strategy itself, characterised by its description, actors, actions, setting, and duration of application.  Secondary outcomes, extracted where reported but not required for inclusion, comprised implementation outcomes (acceptability, adoption, appropriateness, feasibility, fidelity, cost, penetration, and sustainability); service-delivery outcomes (access, coverage, continuity of care, quality indicators, adherence to guidelines, referral completion, and supply-chain performance); and patient-level clinical outcomes (blood-pressure control, glycaemic control, retention in care, treatment adherence, complication rates, and patient experience) | Studies focused solely on NCD epidemiology or clinical effectiveness without describing an implementation or integration strategy. |
| **Study design** | Empirical studies: randomised controlled trials and quasi‑experimental studies; observational designs (cross‑sectional, cohort, interrupted time series); qualitative studies (implementation experiences, barriers, facilitators, acceptability, feasibility); and mixed‑methods studies. | Editorials, commentaries, opinion pieces, and other non‑empirical publications. |
| **Setting** | Primary health care settings: community‑ and district‑level first‑contact facilities (community health posts and centres, district‑level clinics, integrated chronic care platforms, and equivalents), including facilities with a PHC integration component. | Studies conducted exclusively in secondary or tertiary care facilities without a PHC integration component. |
| **Geographic scope** | Studies conducted in sub‑Saharan Africa (World Bank regional classification). | Studies conducted outside sub‑Saharan Africa. |
| **Time period** | Published between 1 January 1990 and 31 December 2025. | Published outside this period. |
| **Language** | Publications in English. | Publications in languages other than English. |
