# Supplementary material for: Implementation strategies for integrating non-communicable disease care into primary healthcare settings in sub-Saharan Africa: A systematic review
Source: PLOS Glob Public Health. 2026 Sep 15;6(9):e0007062. doi: 10.1371/journal.pgph.0007062 (PMC13577421; doi:10.1371/journal.pgph.0007062)
Supplement: Supplementary file 4 [file pgph.0007062.s004.docx]

**Data‑extraction record: eligibility confirmation, data extractors, and extraction date for the included studies.**

**Table Eligibility confirmation and extraction metadata for the 46 included studies.**

| **Title** | **Authors** | **Year** | **Country/Setting** | **Eligible for inclusion** | **Main Findings** | **Sample Size** | **Extracted by** | **Extraction date** |
| --- | --- | --- | --- | --- | --- | --- | --- | --- |
| A mixed methods approach to exploring the moderating factors of implementation fidelity of the integrated chronic disease management model in South Africa | Limakatso Lebina, Tolu Oni, Olufunke A. Alaba & Mary Kawonga | 2022 | South Africa | **Yes** | Moderating factors included facilitation strategies (training, mentorship), intervention complexity, and participant responsiveness. TB patients sharing spaces with NCD/HIV patients compromised fidelity. Poor adherence to one component affected other components. Supply chain, infrastructure, staffing and caseloads were contextual factors | 30 HCWs from 4 PHC facilities in 2 health districts | JO, TYB | 1 May 2026 |
| Acceptability of patient education for hypertension self-management among healthcare providers and beneficiaries in South Africa, 2024: A qualitative study | Simelane X, Kagura J, Nyatela A, Lalla-Edward ST | 2025 | South Africa | **Yes** | Facilitators of acceptability: simple language, knowledge empowerment, and perceived health improvements. Barriers: emotional discomfort from booklet colours, diagnosis denial, and staff shortages. Ongoing training was the preferred strategy to enhance acceptability | 18 HCPs + 13 PLWH+HTN = 31 participants | JO, TYB | 1 May 2026 |
| Alignment between Chronic Disease Policy and Practice: Case Study at a Primary Care Facility | Draper CA, Draper CE, Bresick GF | 2014 | South Africa | **Yes** | Significant gap between policy and implementation. Key contributor was poor policy knowledge among clinicians, leading to an individual rather than team approach. Poor interaction between facility- and community-based services. Staff-related factors were the predominant barrier to policy implementation | 10 individual interviews, 8 focus groups (clinical staff and provincial stakeholders) | JO, TYB | 1 May 2026 |
| Barriers and enablers to improving integrated primary healthcare for non-communicable diseases and mental health conditions in Ethiopia: a mixed methods study | Bekele A, Alem A, Seward N, Eshetu T, Gebremariam TH, Getachew Y, Mengiste W, Medhin G, Fairall L, Sevdalis N, Prince M, Fekadu A, Hanlon C | 2024 | Ethiopia | **Yes** | Four domains identified: training/implementation, awareness/patient needs, resource constraints, and care pathway bottlenecks. Non-availability of resources (medications, laboratory reagents) was the key barrier. Social determinants of health outside health system scope were a substantial challenge. High ORIC scores suggested social desirability bias | 11 qualitative interviewees + 124 health workers completing ORIC questionnaire; 18 health facilities | JO, TYB | 1 May 2026 |
| Barriers to the provision of non-communicable disease care in Zimbabwe: a qualitative study of primary health care nurses | Kamvura TT, Dambi JM, Chiriseri E, Turner J, Verhey R, Chibanda D | 2022 | Zimbabwe | **Yes** | Four cross-cutting barriers identified: poor access to medication and equipment, high cost of private care, poor working conditions, and poor awareness from patients and communities. Community empowerment and task-shifting via the Friendship Bench were proposed as low-cost solutions | 10 general nurses from 5 busy urban clinics | JO, TYB | 1 May 2026 |
| Capacity building for cancer prevention and early detection in the Ugandan primary healthcare facilities: Working toward reducing the unmet needs of cancer control services | Jatho A, Mugisha NM, Kafeero J, Holoya G, Okuku F, Niyonzima N, Orem J | 2020 | Uganda | **Yes** | 488 PHC workers from 118 districts trained; 46 HCWs further trained in cervical, breast, and prostate cancer screening; 32,800 cancer information materials distributed; 75% of districts implemented at least one agreed action at follow-up | 488 PHC workers from 118 districts | JO, TYB | 1 May 2026 |
| Community-Based Participatory Research and System Dynamics Modeling for Improving Retention in Hypertension Care | Ye J, Orji IA, Birkett MA, Hirschhorn LR, Walunas TL, Smith JD, Kandula NR, Shedul GL, Huffman MD, Ojji DB | 2024 | Nigeria | **Yes** | HCW training was the most effective single strategy (retention 29.7% at 12 months). Integrated intervention packages produced the greatest improvements overall (retention 72.4% at 12 months, 68.1% at 24 months, 67.1% at 36 months) | 16 stakeholders (patients, CHWs, nurses, pharmacists, researchers, administrators, policymakers, physicians) | JO, TYB | 1 May 2026 |
| Early Experiences in the Integration of Noncommunicable Diseases into Emergency Primary Health Care, Beni Region, Democratic Republic of the Congo | Ratnayake R, Wittcoff A, Majaribu J, Nzweve JP, Katembo L, Kasonia K, Nzanzu AK, Kiapi L, Ngoy P | 2020 | Democratic Republic of Congo | **Yes** | Consultations for hypertension and diabetes increased significantly before the onset of conflict. 45.4% of hypertension patients demonstrated treatment adherence. Diabetes care remained fragmented. Programme and management costs: USD115 per person per treatment course. NCD can be well-organized through clinical training and cohort analysis, and adherence can be addressed using patient-held cards and monitoring by community health workers | 833 patients (88.7% hypertensives, 95.9% newly diagnosed) | JO, TYB | 1 May 2026 |
| Educational Outreach with an Integrated Clinical Tool for Nurse-Led Non-communicable Chronic Disease Management in Primary Care in South Africa: A Pragmatic Cluster Randomised Controlled Trial | Fairall LR, Folb N, Timmerman V, Lombard C, Steyn K, Bachmann MO, Bateman ED, Lund C, Cornick R, Faris G, Gaziano T, Georgeu-Pepper D, Zwarenstein M, Levitt NS | 2016 | South Africa | **Yes** | Educational outreach was feasible and safe but was not associated with treatment intensification (hypertension, diabetes, respiratory disease) or improved depression case detection compared to control. No adverse effects of expanded prescribing scope observed. Intervention adopted nationally despite lack of superiority | 4393 patients (hypertension n=3227, diabetes n=1842, respiratory n=1157, depression n=2466) across 38 clinics | JO, TYB | 1 May 2026 |
| Empowering Frontline Primary Healthcare Workers in a Global Health Partnership Training of Trainers Intervention to Strengthen Prevention and Control of Cardiovascular Disease in Mozambique | Harris P, Juga E, Bay N, Adams C, Nhatitima P, Mastala A, Matavel N, Mufanequisso A, Mabote N, Mondlane E, Manafe N, Pinto P, Kirkland R, Mazza D, Mocumbe A | 2022 | Mozambique | **Yes** | Three ToT workshops trained 60 mixed cadre healthcare workers in assessment, diagnosis and management of hypertension, diabetes, and cardiovascular risk; of these, 11 became new local trainers. Mean pre- and post-test scores improved in all three training workshops (53% to 90%, 59% to 78%, and 58% to 74% respectively). New local trainers were highly rated by their trainees and reported increased confidence as trainers (mean Likert scale 3.0/5 pre-training to 4.8/5 post-training) | 60 PHC workers; 11 new local trainers | JO, TYB | 1 May 2026 |
| Evaluating smartphone strategies for reliability, reproducibility, and quality of VIA for cervical cancer screening in the Shiselweni region of Eswatini: A cohort study | Asgary R, Staderini N, Mthethwa-Hleta S, Lopez Saavedra PA, Garcia Abrego L, Rusch B, Marie Luce T, Pasipamire LR, Ndlangamandla M, Beideck E, Kerschberger B | 2020 | Eswatini | **Yes** | 4247 patients screened; smartphone mentorship improved nurses' competencies and VIA reliability, reduced false positives, and enabled peer-to-peer education. Agreement rates with expert reviewer: 100% for positives, 95.7% for negatives; Kappa 0.74. HIV and age associated with VIA positivity | 4247 patients screened; 247 reviewed weekly by expert | JO, TYB | 1 May 2026 |
| Evaluating the implementation of group empowerment for people with type 2 diabetes at Grassy Park Community Day Centre, Cape Town: A convergent mixed-methods study | Saban Z, Schouw D, Mash RJ | 2025 | South Africa | **Yes** | 35 patients in 4 groups; 65% of invited patients attended; only 29% attended all 4 sessions; good fidelity to training manual; 30 determinants of successful implementation identified. Incremental costs trivial (R30/month) but opportunity costs higher (R26,252/month) | 35 patients in 4 groups + HCWs (unspecified number) | JO, TYB | 1 May 2026 |
| Evaluation of the effect of the introduction of a hypertension club on the management of hypertension at a community health centre in the Cape Town Metropole | Slingers N, De Villiers PJT | 2009 | South Africa | **Yes** | Limited improvement in patient knowledge after 6 months. 64% collected medication as scheduled. BP >140/90 mmHg reduced from 48% (baseline) to 33% (6 months). Patients satisfied with service but desired shorter waiting times. Benefit seen mainly in establishing the system | 100 patients at inception + 100 patients at 6-month follow-up | JO, TYB | 1 May 2026 |
| Expanding access to non-communicable disease care in rural Malawi: outcomes from a retrospective cohort in an integrated NCD-HIV model | Wroe EB, Kalanga N, Dunbar EL, Nazimera L, Price NF, Shah A, Dullie L, Mailosi B, Gonani G, Ndarama EPL, Talama GC, Bukhman G, Kerr L, Connolly E, Kachimanga C | 2020 | Malawi | **Yes** | 72% for NCD patients. Statistically significant clinical improvement for hypertension, diabetes, asthma, and epilepsy.  The IC3 model, built on an HIV platform, facilitated rapid decentralisation and access to NCD services in rural Malawi | 6233 patients (3334 HIV, 2990 NCD); 14 PHC facilities | JO, TYB | 1 May 2026 |
| Human-centered design as a guide to intervention planning for non-communicable diseases: the BIGPIC study from Western Kenya | Leung CL, Naert M, Andama B, Dong R, Edelman D, Horowitz C, Kiptoo P, Manyara S, Matelong W, Matini E, Naanyu V, Nyariki S, Pastakia S, Valente T, Fuster V, Bloomfield GS, Kamano J, Vedanthan R | 2020 | Kenya | **Yes** | Integrated microfinance-group medical visit model developed; benefits included medication availability, financial resources, peer support, and reduced caregiver burden. Iterative feedback led to context-appropriate modifications. HCD enabled contextually relevant intervention design | Design team + pilot participants (unspecified); FGDs with pilot participants and community members | JO, TYB | 1 May 2026 |
| Implementation of HPV-based screening in Burkina Faso: lessons learned from the PARACAO hybrid-effectiveness study | Mensah K, Kabore C, Zeba S, Bouchon M, Duchesne V, Pourette D, DeBeaudrap P, Dumont A | 2021 | Burkina Faso | **Yes** | 94% screening completeness; only 26% completed screening in a single visit as planned. 95% of women highly satisfied post-result disclosure vs. 65% post-sampling. Good understanding of results improved completeness; time to result disclosure decreased satisfaction. Adaptations made to HCW workload | 350 women in cohort | JO, TYB | 1 May 2026 |
| Implementation outcomes from the Hypertension Treatment in Nigeria program: results from a type 2 hybrid interrupted time series trial | Ye J, Hirschhorn LR, Baldridge AS, Jamro EL, Orji IA, Shedul GL, Ripiye NR, Ojo TM, Eze H, Shedul GJ, Ugwuneji EN, Okoli RCB, Ale BM, Osagie S, Sanuade OA, Iyer G, Kandula NR, Ojji DB, Huffman MD | 2025 | Nigeria | **Yes** | Fixed-dose combination (FDC) drug prescription increased from 16.3% to 65.2%. 95% of PHCs had at least one 30-day supply of BP medication in stock after drug revolving fund implementation. Patient retention at 6 months improved from 59.9% to 63.1% | 21,922 patients; 60 PHCs (78.3% rural) | JO, TYB | 1 May 2026 |
| Implementation, interrupted: Identifying and leveraging factors that sustain after a programme interruption | Hennein R, Ggita J, Ssuna B, Shelley D, Akiteng AR, Davis JL, Katamba A, Armstrong-Hough M | 2021 | Uganda | **Yes** | DM screening ceased due to national supply chain disruption for glucose test strips, which had cascading effects on clinic capacity and clinician self-efficacy. However, culture, compatibility, and clinicians' beliefs about DM screening sustained throughout the interruption | 9 clinic staff; TB clinics in Uganda | JO, TYB | 1 May 2026 |
| Implementing Prevention Interventions for Non-Communicable Diseases within the Primary Health Care System in the Federal Capital Territory, Nigeria | Okpetu EI, Abimbola S, Koot JAR, Kane S | 2018 | Nigeria | **Yes** | Multiple implementation shortfalls identified: insufficient recognition of NCD burden, poor resourcing, poor guidelines operationalisation, inadequate HRH, weak information systems, and poor referral linkages between PHC and secondary/tertiary care. PHC capacity for NCD prevention is weak | Multiple stakeholders (FGD participants, interviewees, observed PHC facilities in FCT) | JO, TYB | 1 May 2026 |
| Improving foot self-care in people with diabetes in Ghana: A development and feasibility randomised trial of a context appropriate, family-orientated diabetic footcare intervention | Suglo JN, Winkley K, Sturt J | 2024 | Ghana | **Yes** | All recruitment, retention, data quality, and randomisation progression criteria met. Intervention was culturally adapted for local literacy and preferences. At 12 weeks post randomisation, efficacy signals favoured the intervention group on improved footcare behaviour, foot self-care efficacy, diabetes knowledge and reduced diabetes distress | 15 (Phase 1 KIIs) + 50 dyads/100 participants (Phase 2 RCT) | JO, TYB | 1 May 2026 |
| Improving integrated depression and non-communicable disease care in Malawi through engaged leadership and supportive implementation climate | Sansbury GM, Pence BW, Zimba C, Yanguela J, Landrum K, Matewere M, Mbota M, Malava JK, Tikhiwa H, Morrison AM, Akiba CF, Gaynes BN, Udedi M, Hosseinipour MC, Stockton MA | 2023 | Malawi | **Yes** | Engaged leadership fuelled positive implementation climate. Effective leaders were approachable and involved in daily operations. Leadership provided mentorship, educated providers on mental health importance, and promoted teamwork and collective ownership. Different leadership levels used their standing to influence provider attitudes | 39 participants across 10 NCD clinics (4 participants per clinic, minus 1 DMO serving 2 clinics) | JO, TYB | 1 May 2026 |
| Integrating gestational diabetes and type 2 diabetes care into primary health care: Lessons from prevention of mother-to-child transmission of HIV in South Africa - A mixed methods study | Mutabazi JC, Enok Bonong PR, Trottier H, Ware LJ, Norris SA, Murphy K, Levitt N, Zarowsky C | 2021 | South Africa | **Yes** | GDM screening not integrated into PMTCT; provincial documents emphasised PMTCT but not GDM or T2DM prevention. PMTCT coverage increased annually. All 30 respondents favoured integrating GDM screening into PMTCT. Western Cape's PMTCT experience suggests integration is feasible | 30 interviewees (HIV/PMTCT experts, HCWs, women with HIV and GDM) + longitudinal PMTCT data across Western Cape | JO, TYB | 1 May 2026 |
| Management of Depression in Chronic Care Patients Using a Task-Sharing Approach in a Real-World Primary Health Care Setting in South Africa: Outcomes of a Cohort Study | Kathree T, Bachmann M, Bhana A, Grant M, Mntambo N, Gigaba S, Kemp CG, Rao D, Petersen I | 2023 | South Africa | **Yes** | Referral for treatment was independently associated with substantial improvements in depression symptoms at 3 months. Study confirms viability of task-shared stepped-up collaborative care for depression using co-located counselling in underserved real-world PHC settings | Adult PHC patients with chronic conditions screened using PHQ-9 across 10 PHC facilities | JO, TYB | 1 May 2026 |
| Measuring the impact of community-based interventions on type 2 diabetes control during the COVID-19 pandemic in Cape Town - A mixed methods study | David NJ, Bresick G, Moodaley N, von Pressentin KB | 2022 | South Africa | **Yes** | Statistically significant relationship between home delivery and improved diabetic control (p<0.01). Mean glycaemic control suboptimal in both groups. 83 interviews confirmed high acceptability of home delivery intervention. Illustrated versatility of community-oriented primary care during pandemic | 461 patients (331 home delivery, 130 non-home delivery) + 83 qualitative interview participants | JO, TYB | 1 May 2026 |
| Medium-to-long term sustainability of a health systems intervention to improve service readiness and quality of NCD patient care and experience at primary care settings in Uganda | Katende D, Kasamba I, Sekitoleko I, Nakuya K, Kusilika C, Buyinza A, Mubiru MC, Mutungi G, Nyirenda M, Grosskurth H, Baisley K | 2023 | Uganda | **Yes** | Mean facility performance (FPS) slightly lower in 2020 vs. 2016 (70.2 vs. 74.8; p=0.18). QoCE declined slightly but 88.2% of patients received adequate quality care. Both service availability and readiness and quality of HT/DM care well preserved 4 years post-trial despite interrupted supplies and reduced supervision | 222 HCWs (131 in 2016, 91 in 2020); 420 patients (88 in 2016, 332 in 2020); 22 health facilities | JO, TYB | 1 May 2026 |
| Minimizing Delays in the Breast Cancer Pathway by Integrating Breast Specialty Care Services at the Primary Health Care Level in Zambia | Songiso M, Pinder LF, Munalula J, Cabanes A, Rayne S, Kapambwe S, Shibemba A, Parham GP | 2020 | Zambia | **Yes** | 1790 symptomatic women attended; 176 (10%) had indications for histologic evaluation; 112 (64%) had pathologic findings compatible with primary breast cancer; 37% early-stage (I/II). Surgery for early-stage cancers performed within 2 weeks of pathologic diagnosis.  Breast care specialty services for symptomatic women were established in a district-level hospital in a resource-constrained setting in Africa | 1790 symptomatic women | JO, TYB | 1 May 2026 |
| Organisational culture and the integrated chronic diseases management model implementation fidelity in South Africa: a cross-sectional study | Lebina L, Kawonga M, Alaba O, Khamisa N, Otwombe K, Oni T | 2020 | South Africa | **Yes** | Overall mean DOC score 3.63/5. Strategies include; Involvement trait highest (3.71), followed by adaptability (3.62) and consistency (3.56). No statistically significant differences in cultural scores between PHC clinics, but scores significantly higher in West Rand district. Leadership intervention needed to enhance adaptability and consistency | 90 staff members across 6 PHC clinics | JO, TYB | 1 May 2026 |
| Pensions and Low Sodium Salt: A Qualitative Evaluation of a New Strategy for Managing Hypertension in Rural South Africa | Lloyd-Sherlock P, Gomez-Olive FX, Ngobeni S, Wagner RG, Tollman S | 2018 | South Africa | **Yes** | 19/20 participants sustained engagement over 3 months. High acceptance of low sodium salt with repeat usage. Strong stakeholder support and willingness to collaborate. Participants reported abatement of hypertension-associated symptoms, supported by BP readings. Intervention deemed feasible for larger-scale testing | 20 participants aged >=60 years | JO, TYB | 1 May 2026 |
| Primary health care response to noncommunicable diseases: an assessment of Wellness Clinics in Ghana | Kushitor MK, William J, Larbi-Sarpong DE, Ampomah MA, Adoma PO, Brightson KTC, Kushitor SB | 2024 | Ghana | **Yes** | All 3 Wellness Clinics were sub-units under the OPD; created to respond to rising NCDs and meet performance review requirements; approximately 300 clients per week at facility level; only 1 of 3 WCs provided community-level NCD prevention; integrated NCD care provided despite health system and individual challenges | 12 healthcare professionals + 26 clients across 3 hospitals | JO, TYB | 1 May 2026 |
| Professional nurses' perceptions and experiences with the implementation of an integrated chronic care model at primary healthcare clinics in South Africa | Mahomed OH, Asmall S | 2017 | South Africa | **Yes** | 264 nurses participated. Prior to implementation: 34% perceived model as an added programme; 36% experienced increased workload. After implementation: nurses reported improved process of care, better patient interaction, improved knowledge and teamwork, and higher work environment satisfaction | 264 professional nurses across 42 PHC facilities | JO, TYB | 1 May 2026 |
| Qualitative evaluation of primary care providers experiences of a training programme to offer brief behaviour change counselling on risk factors for non-communicable diseases in South Africa | Malan Z, Mash R, Everett-Murphy K | 2015 | South Africa | **Yes** | Confidence in counselling improved. Some believed time constraints could be overcome. Significant barriers remained: understaffing, lack of facility support, poor continuity of care. Organisational culture not congruent with patient-centred BBCC approach. Training should be incorporated into undergraduate curricula and CPD | 12 participants in IDIs (nurses and doctors) | JO, TYB | 1 May 2026 |
| Scaling up integrated care for HIV and other chronic conditions in routine health care settings in sub-Saharan Africa: Field notes from Uganda | Moyo F, Birungi J, Garrib A, Namakoola I, Okebe J, Kivuyo S, Mutungi G, Mfinanga S, Nyirenda M, Jaffar S | 2023 | Uganda | **Yes** | Findings suggest that this model of care will be popular among health care providers and policy makers, particularly when they can quantify the benefits that could accrue from this model of care, such as optimal utilization of health resources and reduction of costs associated with health care delivery | - | JO, TYB | 1 May 2026 |
| Screening for diabetic retinopathy in primary care with a mobile fundal camera - evaluation of a South African pilot project | Mash B, Powell D, du Plessis F, van Vuuren U, Michalowska M, Levitt N | 2007 | South Africa | **Yes** | 400 patients screened; 84% had significantly reduced visual acuity; 63% had retinopathy (22% severe non-proliferative, 6% proliferative, 15% maculopathy); 27% required referral for cataracts. A single technician could photograph approximately 10,000 patients per year. Screening with fundal camera feasible in South Africa public sector | 400 diabetic patients at 3 community health centres | JO, TYB | 1 May 2026 |
| Setting up a nurse-led model of care for management of hypertension and diabetes mellitus in a high HIV prevalence context in rural Zimbabwe: a descriptive study | Frieden M, Zamba B, Mukumbi N, Mafaune PT, Makumbe B, Irungu E, Moneti V, Isaakidis P, Garone D, Prasai M | 2020 | Zimbabwe | **Yes** | 3094 patients registered (188 DM only, 2473 HTN only, 433 both). Nurses in 7 PHC facilities and 1 hospital developed competency for DM/HTN diagnosis, treatment initiation, and monitoring. lessons learned from our experience include: the value of POC devices in the management of diabetes; the pressure on services of the added caseload, exacerbated by the availability of free medications in supported health facilities; and the importance of leadership in the successful implementation of care in health facilities | 3094 patients; 9 PHC clinics + 2 hospitals | JO, TYB | 1 May 2026 |
| Stakeholder perspectives to inform the implementation of a community health worker-delivered home management of hypertension intervention in Zimbabwe | Mundagowa PT, Musariri M, Magande P, Hlabangana T, Mukwambo LJ, Zambezi P, Muchemwa-Mutseyekwa P, Mukora-Mutseyekwa F | 2024 | Zimbabwe | **Yes** | Barriers: staff shortage, patient privacy/confidentiality, limited antihypertensive medication access, CHW incentivisation, equipment shortage, and patient health beliefs. Facilitators: leadership commitment, CHW training and support, regular CHW-provider engagement, community partnerships, and CHW self-efficacy and skills | 38 participants (25 CHWs, 10 nurses, 3 health administrators) across 5 PHC facilities | JO, TYB | 1 May 2026 |
| Strategies for supporting the implementation of a task-shared psychological intervention in South Africa's chronic disease services: qualitative insights from health managers' experiences of project MIND | Brooke-Sumner C, Petersen-Williams P, Sorsdahl K, Kruger J, Mahomed H, Myers B | 2022 | South Africa | **Yes** | Four themes mapped: (1) relational work with staff to promote support and reduce resistance; (2) need for staff reorientation and upskilling; (3) strengthening health-social service sector linkages; (4) ongoing monitoring and communication about impacts. Managers' leadership role is critical for scale-up | Health managers from urban and rural PHC facilities in Western Cape (FGDs + IDIs) | JO, TYB | 1 May 2026 |
| Sustainability of integrated hypertension and diabetes with HIV care for people living with HIV at primary health care in South Ethiopia: implication for integration | Badacho AS, Mahomed OH | 2023 | Ethiopia | **Yes** | Overall mean sustainability score: 43.74 (95% CI 42.15-45.33); all facilities scored <55 (threshold for sustainable). Perceived benefits, adaptability, and alignment with organisational goals promoted sustainability. Lack of progress monitoring system, inadequate staff training and involvement, insufficient leadership engagement, and infrastructure limitations were barriers | HIV care and NCD team members from 5 PHC facilities in South Ethiopia | JO, TYB | 1 May 2026 |
| Sustainability of the integrated chronic disease management model at primary care clinics in South Africa | Mahomed OH, Asmall S, Voce A | 2016 | South Africa | **Yes** | Bushbuckridge had highest mean sustainability (71.79), followed by West Rand (70.25) and Dr Kenneth Kaunda (66.50). 11% of facilities scored <55. Clinical leadership involvement, staff behaviour towards ICDM, flexibility of the model, and infrastructure limitations identified as potential negative factors for sustainability | 37 PHC clinics across 3 districts in 3 provinces | JO, TYB | 1 May 2026 |
| Task Shifting the Management of Non-Communicable Diseases to Nurses in Kibera, Kenya: Does It Work? | Some D, Edwards JK, Reid T, Van den Bergh R, Kosgei RJ, Wilkinson E, Baruani B, Kizito W, Khabala K, Shah S, Kibachio J, Musembi P | 2016 | Kenya | **Yes** | 725 nurse consultations met inclusion criteria. Adherence to screening questions 65-86% (average 69%). Weight and BP measurements completed in 89% and 96% of required cases. Lab results reviewed in 91% of indicated visits. Only 17 (2%) consultations referred back to clinical officers. Nurses demonstrated capacity to manage stable NCD patients based on clear and standardized protocols and guidelines, thus paving the way towards task shifting of NCD care | 616 patients; 725 consultations by nurses across 2 integrated PHC facilities | JO, TYB | 1 May 2026 |
| Task shifting to non-physician clinicians for integrated management of hypertension and diabetes in rural Cameroon: a programme assessment at two years | Labhardt ND, Balo JR, Ndam M, Grimm JJ, Manga E | 2010 | Cameroon | **Yes** | All re-assessed facilities had functional sphygmomanometer; 96% stocked antihypertensives. NPC knowledge significantly improved. 796 patients initiated treatment. Retention at 1 year: 18.1%. Systolic BP decreased by 22.8 mmHg (p<0.0001); FPG decreased by 3.4 mmol/L (p<0.001). High patient attrition and low case-detection per NPC limited effectiveness Provision of basic equipment and training in hypertension and diabetes care enhance feasibility | 796 patients initiated; 54 facilities re-assessed (from 69 originally trained) | JO, TYB | 1 May 2026 |
| The comorbidity of HIV, hypertension and diabetes: a qualitative study exploring challenges faced by healthcare providers and patients in selected health facilities where the ICDM model is implemented in South Africa | Godongwana M, De Wet-Billings N, Milovanovic M | 2021 | South Africa | **Yes** | Key challenges: lack of staff capacity, unclear guidelines for HIV chronic comorbidity care, pill burden, non-disclosure, financial burden, poor knowledge of treatments, patient relocation, poor access to treatment, and insufficient integrated chronic programmes. Need for stronger ICDM outcomes and capacity building | 24 participants (HCPs and patients with HIV+HTN or HIV+DM) across 2 provinces | JO, TYB | 1 May 2026 |
| The organisation of primary health care service delivery for non-communicable diseases in Nigeria: A case-study analysis | Ajisegiri WS, Abimbola S, Tesema AG, Odusanya OO, Peiris D, Joshi R | 2022 | Nigeria | **Yes** | Barriers: interprofessional role conflict, perverse incentives sustaining PHC functioning, and perception of PHC as inferior. Enablers: physician presence at PHC and civil society involvement for community linkage. Southern PHCs were better organised to deliver NCD services than northern PHCs | 25 interview participants + 107 FGD participants + 13 PHC facilities observed | JO, TYB | 1 May 2026 |
| Training primary healthcare workers on a task-strengthening strategy for integrating hypertension management into HIV care in Nigeria: implementation strategies, knowledge uptake, and lessons learned | Oladele DA, Odusola AO, Odubela O, Nwaozuru U, Calvin C, Musa Z, Idigbe I, Nwakwo C, Odejobi Y, Aifah A, Kanneh N, Mishra S, Onakomaiya D, Iwelunmor J, Ogedegbe O, Ezechi O | 2023 | Nigeria | **Yes** | Significant improvement in HCW knowledge of hypertension management; mean score improved from 59% (pre-test) to 67.6% (post-test). 75% had good knowledge at baseline. Role-playing and multimedia use improved training uptake. Main barrier: poor delineation of duties; main facilitator: existing task shifting at PHC level | 60 nurses and Community Health Officers (CHOs) | JO, TYB | 1 May 2026 |
| Treatment outcomes among adults with HIV/non-communicable disease multimorbidity attending integrated care clubs in Cape Town, South Africa | Gausi B, Berkowitz N, Jacob N, Oni T | 2021 | South Africa | **Yes** | 247 HIV-infected multimorbid patients enrolled: 89.5% HTN, 8.9% DM+HTN. Adherence maintained (92.2% before vs. 94.2% after IC enrolment). HIV viral suppression: 98.6-99.5%. Optimal BP control: 43.1% to 58.9% to 49.4% across time points. Intensified interventions needed for sustained NCD control | 247 HIV-infected multimorbid adults (HIV+HTN and/or HIV+DM) | JO, TYB | 1 May 2026 |
| Using a nurse-driven and home-based telehealth intervention to improve insulin therapy for people with type 2 diabetes in primary care: a feasibility study | Ngassa Piotie P, Wood P, Muchiri JW, Webb EM, Paul | 2022 | South Africa | **Yes** | HCPs and patients satisfied with intervention; acceptable and appropriate per HCPs; no symptomatic or severe hypoglycaemic events reported. HbA1c lowered by 2.2% (95% CI 1.6-2.8%). Most participants recommended scale-up. Key lesson: high insulin refusal rates need addressing; task sharing and allied HCW involvement can overcome initiation challenges | T2DM patients on maximum oral drugs with suboptimal glycaemic control (HbA1c 9-12%) in City of Tshwane | JO, TYB | 1 May 2026 |
| Using a theory of change to develop an integrated intervention for depression, diabetes and hypertension in Zimbabwe: lessons from the Friendship Bench project | Kamvura TT, Turner J, Chiriseri E, Dambi J, Verhey R, Chibanda D | 2021 | Zimbabwe | **Yes** | ToC singled out the need for expert clients (people with lived experience) for a patient-centred approach. Stakeholders endorsed WhatsApp as an alternative digital platform during COVID-19. Community support groups identified as entry points for NCD care. Final NCD care package framework accepted by policymakers for piloting in 5 geographical areas | 18 stakeholders (grandmothers n=4, policymakers n=2, people with lived experience n=4, HCWs n=2, traditional healers n=2, others) | JO, TYB | 1 May 2026 |

Protocol registered with PROSPERO (CRD420261354803) on 8 April 2026; last search run on 15 April 2026. *JO* and *TYB* denote the two independent data extractors
