# Supplementary material for: Implementation strategies for integrating non-communicable disease care into primary healthcare settings in sub-Saharan Africa: A systematic review
Source: PLOS Glob Public Health. 2026 Sep 15;6(9):e0007062. doi: 10.1371/journal.pgph.0007062 (PMC13577421; doi:10.1371/journal.pgph.0007062)
Supplement: Supplementary file 5 [file pgph.0007062.s005.docx]

**QUALITY ASSESSMENT OF INCLUDED PAPERS**

| **Study** | **Design category** | **C1** | **C2** | **C3** | **C4** | **C5** | **Grade** |
| --- | --- | --- | --- | --- | --- | --- | --- |
| Fairall et al. | Cluster RCT | 1 | 1 | 1 | 1 | 1 | High |
| Lebina et al. | Mixed methods | 1 | 1 | 1 | 0 | 1 | Moderate |
| Bekele et al. | Mixed methods | 1 | 1 | 1 | 1 | 1 | High |
| Ye et al. | Mixed methods | 1 | 0 | 1 | 0 | 1 | Moderate |
| Ratnayake et al. | Mixed methods | 1 | 1 | 1 | 0 | 1 | Moderate |
| Saban et al. | Mixed methods | 1 | 1 | 1 | 1 | 1 | High |
| Leung et al. | Mixed methods | 1 | 0 | 1 | 0 | 1 | Moderate |
| Mensah et al. | Mixed methods | 1 | 1 | 1 | 1 | 1 | High |
| Okpetu et al. | Mixed methods | 1 | 1 | 1 | 0 | 1 | Moderate |
| Mutabazi et al. | Mixed methods | 1 | 1 | 1 | 1 | 1 | High |
| David et al. | Mixed methods | 1 | 1 | 1 | 1 | 1 | High |
| Lloyd-Sherlock et al. | Mixed methods | 1 | 0 | 1 | 0 | 1 | Moderate |
| Ajisegiri et al. | Mixed methods | 1 | 1 | 1 | 1 | 1 | High |
| Oladele et al. | Mixed methods | 1 | 1 | 1 | 0 | 1 | Moderate |
| Ngassa Piotie et al. | Mixed methods | 1 | 0 | 1 | 0 | 1 | Moderate |
| Simelane et al. | Qualitative | 1 | 1 | 1 | 1 | 1 | High |
| Draper et al. | Qualitative | 1 | 1 | 1 | 1 | 1 | High |
| Kamvura et al. | Qualitative | 1 | 1 | 1 | 1 | 1 | High |
| Hennein et al. | Qualitative | 1 | 1 | 1 | 1 | 1 | High |
| Sansbury et al. | Qualitative | 1 | 1 | 1 | 1 | 1 | High |
| Kushitor et al. | Qualitative | 1 | 1 | 1 | 1 | 1 | High |
| Malan et al. | Qualitative | 1 | 1 | 1 | 1 | 1 | High |
| Moyo et al. | Qualitative | 1 | 0 | 1 | 0 | 1 | Moderate |
| Mundagowa et al. | Qualitative | 1 | 1 | 1 | 1 | 1 | High |
| Brooke-Sumner et al. | Qualitative | 1 | 1 | 1 | 1 | 1 | High |
| Godongwana et al. | Qualitative | 1 | 1 | 1 | 1 | 1 | High |
| Kamvura et al. | Qualitative | 1 | 1 | 1 | 1 | 1 | High |
| Songiso et al. | Quantitative descriptive | 1 | 1 | 1 | 0 | 1 | Moderate |
| Lebina et al. | Quantitative descriptive | 1 | 1 | 1 | 0 | 1 | Moderate |
| Mahomed & Asmall | Quantitative descriptive | 1 | 1 | 1 | 0 | 1 | Moderate |
| Mash et al. | Quantitative descriptive | 1 | 1 | 1 | 0 | 1 | Moderate |
| Frieden et al. | Quantitative descriptive | 1 | 1 | 1 | 0 | 1 | Moderate |
| Badacho & Mahomed | Quantitative descriptive | 1 | 1 | 1 | 0 | 1 | Moderate |
| Mahomed et al. | Quantitative descriptive | 1 | 1 | 1 | 0 | 1 | Moderate |
| Some et al. | Quantitative descriptive | 1 | 1 | 1 | 0 | 1 | Moderate |
| Jatho et al. | Quantitative non-randomised | 1 | 1 | 1 | 0 | 1 | Moderate |
| Harris et al. | Quantitative non-randomised | 1 | 0 | 1 | 0 | 1 | Moderate |
| Asgary et al. | Quantitative non-randomised | 1 | 1 | 1 | 0 | 1 | Moderate |
| Slingers & De Villiers | Quantitative non-randomised | 1 | 0 | 1 | 0 | 0 | Low |
| Wroe et al. | Quantitative non-randomised | 1 | 1 | 1 | 1 | 1 | High |
| Ye et al. | Quantitative non-randomised | 1 | 1 | 1 | 1 | 1 | High |
| Kathree et al. | Quantitative non-randomised | 1 | 1 | 1 | 1 | 1 | High |
| Katende et al. | Quantitative non-randomised | 1 | 1 | 1 | 1 | 1 | High |
| Labhardt et al. | Quantitative non-randomised | 1 | 1 | 1 | 1 | 1 | High |
| Gausi et al. | Quantitative non-randomised | 1 | 1 | 1 | 0 | 1 | Moderate |
| Suglo et al. | RCT (feasibility) | 1 | 0 | 1 | 0 | 1 | Moderate |
