# Supplementary material for: Implementation strategies for integrating non-communicable disease care into primary healthcare settings in sub-Saharan Africa: A systematic review
Source: PLOS Glob Public Health. 2026 Sep 15;6(9):e0007062. doi: 10.1371/journal.pgph.0007062 (PMC13577421; doi:10.1371/journal.pgph.0007062)
Supplement: Supplementary file 6 [file pgph.0007062.s006.docx]

**Certainty of the evidence: GRADE and GRADE-CERQual assessment**

*Implementation strategies for integrating non-communicable disease care into primary healthcare settings in sub-Saharan Africa: a systematic review*

| **Review finding (sub-theme)** | **Contributing studies (n; design)** | **Approach** | **Methodological limitations (risk of bias)** | **Coherence/consistency** | **Adequacy/precision** | **Relevance/directness** | **Overall confidence** |
| --- | --- | --- | --- | --- | --- | --- | --- |
| **Theme 1. Multiple, overlapping implementation strategies are used to integrate NCD care into PHC, with task-shifting / task-sharing and integrated chronic care models the most prominent** | | | | | | | |
| **1.1 Task-shifting and task-sharing to nurses, CHOs and non-physician clinicians** | 7 (Some et al., 2016; Labhardt et al., 2010; Frieden et al., 2020; Oladele et al., 2023; Mahomed & Asmall, 2017; Kathree et al., 2023; Brooke-Sumner et al., 2022). Descriptive/retrospective, before–after, cohort, cross-sectional, qualitative | Both | Minor–moderate. Grades range High (Labhardt et al., 2010; Kathree et al., 2023; Brooke-Sumner et al., 2022) to Moderate; predominantly non-randomised and descriptive designs | Consistent. Delegation of stable NCD care to non-physician cadres feasible with acceptable protocol adherence across all settings | Good. Multi-country (Cameroon, Kenya, South Africa, Zimbabwe, Nigeria); hundreds–thousands of patients | High | **Moderate–High.** Consistent, multi-country evidence; limited chiefly by the predominance of non-randomised designs. |
| **1.2 Integrated chronic care models (ICDM, IC3, HIV–NCD clubs)** | 9 (Wroe et al., 2020; Lebina, Oni, et al., 2020; Mahomed & Asmall, 2017; Mahomed et al., 2016; Godongwana et al., 2021; Gausi et al., 2021; Moyo et al., 2023; Frieden et al., 2020; Badacho & Mahomed, 2023). Cohort, cross-sectional, qualitative, descriptive | Both | Moderate. One High cohort (Wroe et al., 2020) and High qualitative (Godongwana et al., 2021); remainder Moderate cross-sectional/descriptive | Consistent where resourced. Models enabled simultaneous HIV + HTN/DM management; fidelity sensitive to context | Strong. Several distinct models; largest cohort 6,233 patients across 14 facilities | High | **Moderate.** Consistent operational evidence; effect estimates drawn mostly from uncontrolled designs. |
| **1.3 Workforce capacity-building, training and educational outreach** | 6 (Jatho et al., 2020; Harris et al., 2022; Fairall et al., 2016; Oladele et al., 2023; Malan et al., 2015; Mahomed & Asmall, 2017). One cluster RCT; pre–post; qualitative | Both | Moderate. One cluster RCT (Fairall et al., 2016, High) and High qualitative (Malan et al., 2015); training studies mostly single-arm pre–post | Provider knowledge and confidence gains highly consistent; downstream clinical effects mixed (Fairall et al. [2016] showed no superiority) | Good. Includes a national programme (488 workers across 118 districts) | High for knowledge; indirect for patient outcomes | **Moderate.** Consistent knowledge gains; uncertain translation to clinical outcomes and variable durability. |
| **1.4 Digital health and decision-support tools** | 5 (Asgary et al., 2020; Mash et al., 2007; Ngassa Piotie et al., 2022; Mensah et al., 2021; Fairall et al., 2016). Cohort, pilot/feasibility, mixed methods | Both | Moderate. Mensah et al. (2021) and Fairall et al. (2016) High; Asgary et al. (2020), Mash et al. (2007), Ngassa Piotie et al. (2022) Moderate; several feasibility / pilot designs | Consistent improvements in diagnostic quality and standardisation; all at feasibility or pilot level | Moderate. Small pilots (e.g. TIP [Ngassa Piotie et al., 2022] feasibility) alongside larger screening cohorts | High | **Low–Moderate.** Promising and consistent, but dominated by feasibility/pilot designs without controlled comparison. |
| **1.5 Community- and group-based delivery models** | 7 (Leung et al., 2020; Saban et al., 2025; Slingers & De Villiers, 2009; Lloyd-Sherlock et al., 2018; David et al., 2022; Mundagowa et al., 2024; Kamvura et al., 2021). Mixed methods, before–after, pilot, qualitative | Both | Moderate. Includes one Low-grade before–after (Slingers & De Villiers, 2009) and Moderate pilots; Saban et al. (2025), David et al. (2022), Mundagowa et al. (2024), Kamvura et al. (2021) High | Consistent. Improved access, peer support and engagement | Moderate. Several small pilot / feasibility studies | High | **Moderate.** Consistent direction of effect from small, design-limited studies. |
| **1.6 Guidelines, protocols and quality-improvement packages** | 6 (Bekele et al., 2024; Draper et al., 2014; Ye et al., 2025; Ratnayake et al., 2020; Songiso et al., 2020; Okpetu et al., 2018). Mixed methods, qualitative, interrupted time series, descriptive | Both | Minor–moderate. Bekele et al. (2024), Draper et al. (2014), Ye et al. (2025) High; Ratnayake et al. (2020), Songiso et al. (2020), Okpetu et al. (2018) Moderate | Consistent. Structured guidelines, HEARTS and protocols supported standardised care; uptake varied with policy awareness and resourcing | Good. Includes 21,922-patient HEARTS programme | High | **Moderate.** Well-evidenced characterisation of the strategy; heterogeneous real-world uptake. |
| **Theme 2. Barriers and facilitators to integration are predominantly health-system level, with workforce, supply chain, and leadership emerging as decisive** | | | | | | | |
| **2.1 Workforce constraints and competing demands** | 8 (Bekele et al., 2024; Kamvura et al., 2022; Mahomed & Asmall, 2017; Malan et al., 2015; Mundagowa et al., 2024; Godongwana et al., 2021; Brooke-Sumner et al., 2022; Lebina, Oni, et al., 2020). Qualitative and mixed methods | CERQual | Minor. Predominantly High-grade qualitative studies; two Moderate | Strong. Staff shortages and high workload consistently constrain implementation | Rich. Thick qualitative data across five or more countries | High | **High.** Rich, coherent, cross-country qualitative evidence with minor methodological concerns. |
| **2.2 Medication, supply chain and infrastructure** | 8 (Bekele et al., 2024; Kamvura et al., 2022; Hennein et al., 2021; Mundagowa et al., 2024; Okpetu et al., 2018; Frieden et al., 2020; Ye et al., 2025; Ratnayake et al., 2020). Qualitative, operational, interrupted time series | Both | Minor. High qualitative (Bekele et al., 2024; Kamvura et al., 2022; Hennein et al., 2021; Mundagowa et al., 2024) and quantitative (Ye et al., 2025, High; others Moderate) | Strong. Stock-outs recurrent and decisive; drug revolving funds and pre-positioning stabilised supply | Rich. Includes a natural experiment (Hennein et al., 2021, interruption) and operational data (95% availability, Ye et al., 2025) | High | **High.** Highly consistent and decisive across qualitative and operational evidence. |
| **2.3 Leadership, organisational climate and culture** | 6 (Sansbury et al., 2023; Lebina, Kawonga, et al., 2020; Brooke-Sumner et al., 2022; Mahomed et al., 2016; Badacho & Mahomed, 2023; Kamvura et al., 2022). Qualitative + Denison / NHS-SM instruments | Both | Minor–moderate. High qualitative (Sansbury et al., 2023; Brooke-Sumner et al., 2022; Kamvura et al., 2022) with Moderate cross-sectional culture/sustainability studies | Strong. Engaged leadership and supportive culture consistently facilitate; weak leadership impedes | Good. Triangulated qualitative data plus validated instruments (Denison, NHS-SM) | High | **Moderate–High.** Coherent and triangulated; quantitative components mostly cross-sectional. |
| **2.4 Policy alignment and guideline operationalisation** | 6 (Draper et al., 2014; Okpetu et al., 2018; Bekele et al., 2024; Ajisegiri et al., 2022; Mutabazi et al., 2021; Kushitor et al., 2024). Qualitative and mixed-methods case studies | CERQual | Minor. Mostly High (Draper et al., 2014; Bekele et al., 2024; Ajisegiri et al., 2022; Mutabazi et al., 2021; Kushitor et al., 2024); one Moderate (Okpetu et al., 2018) | Strong. Persistent policy–practice gap consistently attributed to weak dissemination, unclear roles and few operational tools | Rich. Multi-country case studies | High | **High.** Consistent and well-evidenced across five countries. |
| **2.5 Patient and community factors** | 7 (Simelane et al., 2025; Suglo et al., 2024; Lloyd-Sherlock et al., 2018; Mundagowa et al., 2024; Godongwana et al., 2021; Kamvura et al., 2022; David et al., 2022). Qualitative, feasibility, mixed methods | Both | Minor–moderate. High (Simelane et al., 2025; Mundagowa et al., 2024; Godongwana et al., 2021; Kamvura et al., 2022; David et al., 2022) with Moderate feasibility studies | Strong. Health literacy, stigma, financial hardship and acceptability shape uptake; peer support and culturally adapted education enhance engagement | Good | High | **Moderate–High.** Coherent and consistent; some data from small feasibility studies. |
| **2.6 Digital infrastructure and connectivity** | 5 (Asgary et al., 2020; Ngassa Piotie et al., 2022; Mash et al., 2007; Mensah et al., 2021; Fairall et al., 2016). Cohort, pilot, mixed methods | Both | Moderate. Mensah et al. (2021), Fairall et al. (2016) High; Asgary et al. (2020), Mash et al. (2007), Ngassa Piotie et al. (2022) Moderate | Consistent. Digital tools extend reach but depend on connectivity, devices and digital literacy | Moderate. Fewer studies; connectivity mostly incidental to the primary digital intervention | High | **Moderate.** Consistent direction but a thinner supporting evidence base. |
| **Theme 3. Implementation strategies improve service delivery and clinical outcomes, but effects are heterogeneous and depend on intervention design and context** | | | | | | | |
| **3.1 Service delivery and access** | 8 (Wroe et al., 2020; Some et al., 2016; Labhardt et al., 2010; Songiso et al., 2020; Frieden et al., 2020; Ratnayake et al., 2020; Mensah et al., 2021; Asgary et al., 2020). Cohort, descriptive, operational | GRADE | Moderate. Mostly cohort / descriptive; Wroe et al. (2020), Labhardt et al. (2010), Mensah et al. (2021) High, remainder Moderate | Consistent. Integration expanded service availability and increased screening / treatment uptake | Moderate. Large denominators but uncontrolled estimates | High | **Moderate.** Consistent multi-site observational evidence; no randomised comparison of access outcomes. |
| **3.2 Provider knowledge and competence** | 6 (Harris et al., 2022; Jatho et al., 2020; Oladele et al., 2023; Mahomed & Asmall, 2017; Malan et al., 2015; Asgary et al., 2020). Pre–post, cross-sectional, qualitative | GRADE | Moderate–serious. Single-arm pre–post without controls (Harris et al., 2022; Jatho et al., 2020; Oladele et al., 2023); Malan et al. (2015) High qualitative | Consistent, often large gains (e.g. mean test scores 53% → 90%) | Moderate | High for knowledge; indirect for patient benefit | **Moderate.** Large, consistent knowledge gains; uncontrolled designs and variable durability lower certainty. |
| **3.3 Clinical outcomes – blood-pressure control** | 6 (Wroe et al., 2020; Labhardt et al., 2010; Slingers & De Villiers, 2009; Ye et al., 2025; Gausi et al., 2021; Ratnayake et al., 2020). Interrupted time series, cohort, before–after | GRADE | Moderate. One High interrupted time series (Ye et al., 2025) and High cohorts (Labhardt et al., 2010; Wroe et al., 2020); one Low (Slingers & De Villiers, 2009) | Consistent improvement in BP control / systolic BP across settings and designs; effect sometimes large (SBP −22.8 mmHg, Labhardt et al., 2010) | Moderate | High | **Moderate.** Consistent, occasionally large effects across designs, but predominantly uncontrolled; rated up from Low on consistency and effect size. |
| **3.4 Clinical outcomes – glycaemic control and retention** | 5 (Wroe et al., 2020; David et al., 2022; Gausi et al., 2021; Ngassa Piotie et al., 2022; Labhardt et al., 2010). Cohort, mixed methods, feasibility | GRADE | Moderate. Mix of High and Moderate designs; some small feasibility samples | Inconsistent. Glycaemic control suboptimal in several models (David et al., 2022; Gausi et al., 2021, ~50%) yet improved in others (TIP [Ngassa Piotie et al., 2022] HbA1c −2.2%; Labhardt et al., 2010) | Moderate–low. Some small samples; retention reported variably | High | **Low.** Inconsistent effects on glycaemic control and retention across uncontrolled designs. |
| **3.5 Patient experience and acceptability** | 7 (Simelane et al., 2025; Suglo et al., 2024; Saban et al., 2025; Lloyd-Sherlock et al., 2018; David et al., 2022; Mahomed & Asmall, 2017; Kushitor et al., 2024). Qualitative and mixed methods | Both | Minor–moderate. High (Simelane et al., 2025; Saban et al., 2025; David et al., 2022; Kushitor et al., 2024) with Moderate feasibility studies | Consistent. Services highly acceptable, with improved interaction, dignity and convenience | Good | High | **Moderate–High.** Consistent and coherent acceptability evidence. |
| **3.6 Implementation outcomes – fidelity, sustainability, cost** | 8 (Lebina, Oni, et al., 2020; Mahomed et al., 2016; Badacho & Mahomed, 2023; Saban et al., 2025; Hennein et al., 2021; Katende et al., 2023; Ratnayake et al., 2020; Mensah et al., 2021). NHS-SM, cross-sectional, qualitative, mixed methods | Both | Moderate. High (Saban et al., 2025; Hennein et al., 2021; Katende et al., 2023; Mensah et al., 2021) with Moderate NHS-SM / cross-sectional studies | Mixed. Short-term fidelity and adoption achievable; long-term sustainability vulnerable to supply and funding interruption; measured inconsistently | Moderate. Heterogeneous instruments (NHS-SM, bespoke tools) | High | **Moderate / Low.** Moderate for short-term fidelity/adoption; Low for long-term sustainability given inconsistent measurement and demonstrated fragility. |
| **Theme 4. Scale-up and sustainability depend on system-level investment, embedded leadership, and integration with existing chronic care platforms** | | | | | | | |
| **4.1 Leveraging the HIV platform for NCD integration** | 6 (Wroe et al., 2020; Gausi et al., 2021; Mutabazi et al., 2021; Badacho & Mahomed, 2023; Moyo et al., 2023; Oladele et al., 2023). Cohort, mixed methods, qualitative | Both | Moderate. High (Wroe et al., 2020; Mutabazi et al., 2021) with Moderate cohort / qualitative studies | Consistent. HIV chronic-care infrastructure a feasible, efficient platform; improved continuity for multimorbid patients | Good. Operational and clinical data across several countries | High | **Moderate.** Consistent multi-country operational evidence; mostly observational. |
| **4.2 Embedded leadership and continuous mentorship** | 6 (Sansbury et al., 2023; Brooke-Sumner et al., 2022; Lebina, Kawonga, et al., 2020; Mahomed et al., 2016; Frieden et al., 2020; Kamvura et al., 2022). Qualitative + sustainability instruments | Both | Minor–moderate. High qualitative with Moderate quantitative components | Strong. Sustained implementation depended on engaged leadership, mentorship and organisational ownership | Good | High | **Moderate–High.** Coherent across settings and convergent with finding 2.3. |
| **4.3 Financing and supply-chain investment** | 5 (Ye et al., 2025; Hennein et al., 2021; Frieden et al., 2020; Ratnayake et al., 2020; Mahomed et al., 2016). Interrupted time series, qualitative, operational | Both | Moderate. Ye et al. (2025) and Hennein et al. (2022) High; remainder Moderate | Consistent. Drug revolving funds, pooled procurement and predictable financing protect gains; episodic donor funding endangers sustainability | Moderate. Includes operational (revolving fund) and natural-experiment (Hennein et al., 2021) evidence | High | **Moderate.** Consistent and mechanistically coherent; limited number of studies. |
| **4.4 Iterative, context-adapted scale-up** | 7 (Leung et al., 2020; Kamvura et al., 2021; Saban et al., 2025; Bekele et al., 2024; Mensah et al., 2021; Ngassa Piotie et al., 2022; Suglo et al., 2024). Human-centred design, theory-of-change, mixed methods, feasibility | Both | Minor–moderate. Several High (Kamvura et al., 2021; Saban et al., 2025; Bekele et al., 2024; Mensah et al., 2021) with Moderate studies | Consistent. Human-centred, theory-informed and iteratively adapted designs improved fit, feasibility and acceptability | Good | High | **Moderate.** Consistent design-level evidence; outcomes mostly feasibility / acceptability. |
| **4.5 Integration with health-system-strengthening priorities** | 7 (Ajisegiri et al., 2022; Okpetu et al., 2018; Kushitor et al., 2024; Draper et al., 2014; Katende et al., 2023; Mahomed & Asmall, 2017; Bekele et al., 2024). Mixed methods, qualitative, cross-sectional | Both | Minor–moderate. Mostly High with Moderate studies | Strong. Effective integration required alignment with workforce, governance, financing, supply chain and information systems within national PHC strategies | Rich | High | **Moderate–High.** Consistent, policy-relevant evidence across several countries. |

**Summary of certainty across the evidence base**

Confidence was **highest** for the findings on barriers and facilitators (Theme 2) – in particular workforce constraints, supply-chain and infrastructure barriers, and the policy–practice gap – which rest on a rich and coherent body of predominantly high-quality qualitative evidence converging across countries. Confidence was **intermediate** for the characterisation of the six strategy clusters (Theme 1), for service-delivery, access, provider-knowledge and acceptability outcomes, and for the scale-up mechanisms (Theme 4), which are supported by consistent but largely observational and descriptive evidence. Confidence was **lowest** for effects on glycaemic control and retention and for long-term sustainability, where findings were inconsistent, measured heterogeneously, and drawn from uncontrolled designs. Notably, certainty for blood-pressure control (Moderate) exceeded that for glycaemic control (Low), reflecting the more consistent and occasionally larger effects observed for hypertension. These gradations should be read alongside the review's conclusions, which are framed as associations rather than causal effects except where supported by the randomised and quasi-experimental studies (Fairall et al., 2016; Ye et al., 2025; Suglo et al., 2024).
