# Supplementary material for: Implementation strategies for integrating non-communicable disease care into primary healthcare settings in sub-Saharan Africa: A systematic review
Source: PLOS Glob Public Health. 2026 Sep 15;6(9):e0007062. doi: 10.1371/journal.pgph.0007062 (PMC13577421; doi:10.1371/journal.pgph.0007062)
Supplement: Supplementary file 7 [file pgph.0007062.s007.docx]

**Thematic synthesis of implementation strategies for integrating NCD care into PHC in sub-Saharan Africa**

| **Main theme** | **Sub-theme** | **Supporting studies** | **Illustrative findings** | **Strength of evidence** |
| --- | --- | --- | --- | --- |
| Multiple, overlapping implementation strategies are used to integrate NCD care into PHC, with task-shifting/task-sharing and integrated care models the most prominent | Task-shifting and task-sharing to nurses and non-physician clinicians | Some et al.; Labhardt et al.; Frieden et al.; Oladele et al.; Mahomed & Asmall; Kathree et al.; Brooke-Sumner et al. | Stable NCD care delegated to nurses, CHOs, and non-physician clinicians improved access and demonstrated acceptable adherence to clinical protocols. | Moderate–High (multi-site evaluations and a cluster RCT; consistent direction of effect). |
|  | Integrated chronic care models | Wroe et al.; Lebina et al.; Mahomed & Asmall; Mahomed et al.; Godongwana et al.; Gausi et al.; Moyo et al.; Frieden et al.; Badacho & Mahomed | ICDM, IC3, and integrated HIV-NCD club models supported simultaneous management of HIV with hypertension and diabetes and produced clinically meaningful improvements where well-resourced. |  |
|  | Workforce capacity-building, training and educational outreach | Jatho et al.; Harris et al.; Fairall et al.; Oladele et al.; Malan et al.; Mahomed & Asmall | Training, cascade ToT, and educational outreach with integrated clinical tools improved provider knowledge and confidence; effects on clinical outcomes were mixed. |  |
|  | Digital health and decision-support tools | Asgary et al.; Mash et al.; Ngassa Piotie et al.; Mensah et al.; Fairall et al. | Smartphone-based mentorship, mobile fundal cameras, telehealth platforms, and HPV screening with electronic tools improved diagnostic quality and standardisation. |  |
|  | Community- and group-based delivery models | Leung et al.; Saban et al.; Slingers & De Villiers; Lloyd-Sherlock et al.; David et al.; Mundagowa et al.; Kamvura et al. | Hypertension and diabetes clubs, group empowerment, CHW home delivery and home-based hypertension management improved access, peer support, and engagement. |  |
|  | Guidelines, protocols and quality-improvement packages | Bekele et al.; Draper et al.; Ye et al.; Ratnayake et al.; Songiso et al.; Okpetu et al. | Implementation of structured guidelines, the WHO HEARTS package, and clinical protocols supported standardised NCD care, although uptake varied with policy awareness and resourcing. |  |
| Barriers and facilitators to integration are predominantly health-system level, with workforce, supply chain, and leadership emerging as decisive | Workforce constraints and competing demands | Bekele et al.; Kamvura et al.; Mahomed & Asmall; Malan et al.; Mundagowa et al.; Godongwana et al.; Brooke-Sumner et al.; Lebina et al. | Staff shortages, high workloads, and competing priorities consistently constrained the implementation of integrated NCD care. | Moderate–High (large body of qualitative and mixed-methods evidence converging across countries). |
|  | Medication, supply chain and infrastructure | Bekele et al.; Kamvura et al.; Hennein et al.; Mundagowa et al.; Okpetu et al.; Frieden et al.; Ye et al.; Ratnayake et al. | Stock-outs of essential medicines and reagents were a recurrent and decisive barrier; drug revolving funds and pre-positioned supplies stabilised availability where established. |  |
|  | Leadership, organisational climate and culture | Sansbury et al.; Lebina et al.; Brooke-Sumner et al.; Mahomed et al.; Badacho & Mahomed; Kamvura et al. | Engaged, approachable leaders and supportive organisational culture were major facilitators; weak leadership and unclear authority impeded implementation. |  |
|  | Policy alignment and guideline operationalisation | Draper et al.; Okpetu et al.; Bekele et al.; Ajisegiri et al.; Mutabazi et al.; Kushitor et al. | Persistent gaps between policy intent and frontline practice were attributable to weak guideline dissemination, unclear roles, and limited operational tools. |  |
|  | Patient and community factors | Simelane et al.; Suglo et al.; Lloyd-Sherlock et al.; Mundagowa et al.; Godongwana et al.; Kamvura et al.; David et al. | Health literacy, stigma, financial hardship, and acceptability shaped uptake; community partnerships, peer support, and culturally adapted education enhanced engagement. |  |
|  | Digital infrastructure and connectivity | Asgary et al.; Ngassa Piotie et al.; Mash et al.; Mensah et al.; Fairall et al. | Digital interventions extended reach but depended on connectivity, device availability, and digital literacy; weak infrastructure constrained scalability. |  |
| Implementation strategies improve service delivery and clinical outcomes, but effects are heterogeneous and dependent on intervention design and context | Service delivery and access | Wroe et al.; Some et al.; Labhardt et al.; Songiso et al.; Frieden et al.; Ratnayake et al.; Mensah et al.; Asgary et al. | Integration expanded NCD service availability and increased screening and treatment uptake at PHC level. | Moderate (multi-site quasi-experimental and cohort evidence; one cluster RCT). |
|  | Provider knowledge and competence | Harris et al.; Jatho et al.; Oladele et al.; Mahomed & Asmall; Malan et al.; Asgary et al. | Training and supportive supervision produced large gains in provider knowledge, confidence, and clinical performance; durability was variable. |  |
|  | Clinical outcomes (BP, glycaemic, retention) | Wroe et al.; Labhardt et al.; Slingers & De Villiers; Ye et al.; Gausi et al.; Ratnayake et al.; Ngassa Piotie et al.; David et al. | Hypertension and diabetes control improved within integrated and task-shifted models; gains were less consistent for diabetes than hypertension and depended on retention. |  |
|  | Patient experience and acceptability | Simelane et al.; Suglo et al.; Saban et al.; Lloyd-Sherlock et al.; David et al.; Mahomed & Asmall; Kushitor et al. | Integrated services were highly acceptable, with reported improvements in patient–provider interaction, dignity, and convenience. |  |
|  | Implementation outcomes (fidelity, sustainability, cost) | Lebina et al.; Mahomed et al.; Badacho & Mahomed; Saban et al.; Hennein et al.; Katende et al.; Ratnayake et al.; Mensah et al. | Fidelity, adoption, and short-term sustainability were achievable; longer-term sustainability was vulnerable to supply-chain and funding interruptions. |  |
| Scale-up and sustainability depend on system-level investment, embedded leadership, and integration with existing chronic care platforms | Leveraging the HIV platform for NCD integration | Wroe et al.; Gausi et al.; Mutabazi et al.; Badacho & Mahomed; Moyo et al.; Oladele et al. | HIV chronic care infrastructure provided a feasible and efficient platform for embedding NCD services and improved continuity for multimorbid patients. | Moderate (consistent multi-country evidence with operational and clinical data). |
|  | Embedded leadership and continuous mentorship | Sansbury et al.; Brooke-Sumner et al.; Lebina et al.; Mahomed et al.; Frieden et al.; Kamvura et al. | Sustained implementation depended on engaged frontline leadership, mentorship, and organisational ownership. |  |
|  | Financing and supply-chain investment | Ye et al.; Hennein et al.; Frieden et al.; Ratnayake et al.; Mahomed et al. | Drug revolving funds, pooled procurement, and predictable financing protected gains; episodic donor funding placed sustainability at risk. |  |
|  | Iterative, context-adapted scale-up | Leung et al.; Kamvura et al.; Saban et al.; Bekele et al.; Mensah et al.; Ngassa Piotie et al.; Suglo et al. | Human-centred, theory-informed, and iteratively adapted designs aligned interventions with local context, supporting feasibility and acceptability. |  |
|  | Integration with health system strengthening priorities | Ajisegiri et al.; Okpetu et al.; Kushitor et al.; Draper et al.; Katende et al.; Mahomed & Asmall; Bekele et al. | Effective integration required alignment with workforce planning, governance, financing, supply chain, and information systems within national PHC strategies. |  |
