# Supplementary material for: Implementation strategies for integrating non-communicable disease care into primary healthcare settings in sub-Saharan Africa: A systematic review
Source: PLOS Glob Public Health. 2026 Sep 15;6(9):e0007062. doi: 10.1371/journal.pgph.0007062 (PMC13577421; doi:10.1371/journal.pgph.0007062)
Supplement: Supplementary file 8 [file pgph.0007062.s008.docx]

**Mapping implementation-strategy clusters to implementation, service delivery, and patient/clinical outcomes.**

| **Strategy cluster** | **Implementation outcomes (Proctor)** | **Service-delivery outcomes** | **Patient / clinical outcomes** |
| --- | --- | --- | --- |
| **1. Task-shifting and task-sharing** | **Feasibility:** nurse/non-physician-clinician-led management feasible; only 2% of consultations referred back to clinicians (Some et al., 2016); NPC programme feasible at two years (Labhardt et al., 2010). **Fidelity:** acceptable adherence to clinical protocols (screening/measurement completed in 65–96% of indicated cases; Some et al., 2016). **Acceptability:** task-shared depression care acceptable and viable (Kathree et al., 2023); training improved acceptance (Oladele et al., 2023). **Adoption:** nurse-led PC101 model adopted nationally (Fairall et al., 2016). **Sustainability:** manager strategies identified to sustain task-shared psychological care (Brooke-Sumner et al., 2022). | **Access:** expanded NCD care in underserved and rural settings (Labhardt et al., 2010; Some et al., 2016; Frieden et al., 2020). **Continuity/retention at PHC:** low referral-out (Some et al., 2016); 3,094 HTN/DM patients registered and monitored (Frieden et al., 2020). | **Hypertension:** systolic BP reduced by 22.8 mmHg (Labhardt et al., 2010). **Diabetes:** fasting plasma glucose reduced by 3.4 mmol/L (Labhardt et al., 2010). **Depression:** symptom improvement at 3 months (Kathree et al., 2023). **Retention:** one-year retention low (18.1%; Labhardt et al., 2010) – a recurrent limitation. |
| **2. Integrated chronic care models** | **Feasibility:** IC3 model feasible with 72% one-year NCD retention (Wroe et al., 2020). **Fidelity:** ICDM fidelity moderated by organisational culture, workload and TB/HIV/NCD co-location (Lebina, Oni, et al., 2020; Lebina, Kawonga, et al., 2020). **Adoption:** ICDM adopted across multiple districts (Mahomed & Asmall, 2017). **Sustainability:** variable – 11% of ICDM facilities below the NHS-SM threshold (Mahomed et al., 2016); all facilities below threshold in South Ethiopia (Badacho & Mahomed, 2023). | **Access/coverage:** expanded NCD service availability (Wroe et al., 2020: 6,233 patients across 14 facilities). **Continuity:** improved through co-located HIV + NCD visits (Gausi et al., 2021; Moyo et al., 2023). | **Multiple conditions:** significant clinical improvement for HTN, DM, asthma and epilepsy (Wroe et al., 2020). **Hypertension/HIV:** BP control 43→59→49% across time points; HIV viral suppression maintained at 98–99% (Gausi et al., 2021). **Diabetes:** glycaemic control suboptimal (~50% optimal; Gausi et al., 2021). |
| **3. Workforce capacity-building, training and educational outreach** | **Adoption:** 75% of districts implemented ≥1 agreed action at follow-up (Jatho et al., 2020); PC101 adopted nationally (Fairall et al., 2016). **Feasibility:** educational outreach feasible and safe (Fairall et al., 2016); cascade training-of-trainers feasible (Harris et al., 2022). **Fidelity:** training delivered as designed; new local trainers certified (Harris et al., 2022). | **Provider knowledge/competence:** large gains – mean test scores 53→90% (Harris et al., 2022); 59→67.6% (Oladele et al., 2023); 488 workers trained across 118 districts (Jatho et al., 2020); improved counselling capability (Malan et al., 2015). | **Direct clinical effect limited:** PC101 not superior to the active comparator for treatment intensification or depression detection (Fairall et al., 2016). **Indirect:** clinical benefit inferred via improved provider competence rather than measured directly. |
| **4. Digital health and decision-support tools** | **Feasibility:** mobile fundal camera feasible (~10,000 patients per technician per year; Mash et al., 2007); smartphone mentorship feasible (Asgary et al., 2020). **Acceptability:** telehealth insulin support acceptable and safe, with no severe hypoglycaemia (Ngassa Piotie et al., 2022); 95% of women satisfied post-result (Mensah et al., 2021). **Fidelity:** high image-review agreement with expert (100%/95.7%; Kappa 0.74; Asgary et al., 2020). | **Diagnostic quality:** improved quality and standardisation of screening (Asgary et al., 2020; Mash et al., 2007). **Screening coverage:** 94% screening completeness (Mensah et al., 2021); 4,247 women screened (Asgary et al., 2020). | **Diabetes:** HbA1c reduced by 2.2% (Ngassa Piotie et al., 2022). **Retinopathy:** 63% detected and 27% referred for care (Mash et al., 2007). **Cervical screening:** completion and result disclosure improved (Mensah et al., 2021). |
| **5. Community- and group-based delivery models** | **Feasibility:** 19/20 older adults retained over 3 months (Lloyd-Sherlock et al., 2018); human-centred-design model feasible (Leung et al., 2020). **Acceptability:** home delivery highly acceptable (David et al., 2022); low-sodium salt accepted with repeat use (Lloyd-Sherlock et al., 2018). **Fidelity/reach:** good fidelity to the training manual; 65% attended ≥1 session but only 29% attended all sessions (Saban et al., 2025). | **Access/continuity:** home and community delivery expanded access and preserved continuity during COVID-19 (David et al., 2022; Mundagowa et al., 2024). | **Hypertension:** proportion with BP >140/90 reduced 48→33% at 6 months (Slingers & De Villiers, 2009). **Diabetes:** glycaemic control improved but remained suboptimal (David et al., 2022). **Self-care:** foot-care behaviour and self-efficacy improved (Suglo et al., 2024); high patient experience across studies. |
| **6. Guidelines, protocols and quality-improvement packages** | **Adoption/fidelity:** fixed-dose-combination prescribing 16.3→65.2% under WHO HEARTS (Ye et al., 2025); guideline uptake varied with awareness and resourcing (Bekele et al., 2024; Okpetu et al., 2018). **Feasibility:** NCD integration into emergency PHC is feasible at ~USD 115 per person (Ratnayake et al., 2020). **Penetration:** breast-specialty services established at PHC level (Songiso et al., 2020). | **Medication availability:** 95% of PHCs stocked ≥30-day BP supply after a drug revolving fund (Ye et al., 2025). **Access:** 1,790 women seen with early cancer detection (Songiso et al., 2020); HTN/DM consultations increased (Ratnayake et al., 2020). | **Hypertension:** 6-month retention 59.9→63.1% (Ye et al., 2025). **Cancer:** 64% of referred women had confirmed cancer; early-stage surgery within 2 weeks (Songiso et al., 2020). **Adherence:** 45.4% of HTN patients were adherent (Ratnayake et al., 2020). |

Implementation outcomes are classified using the taxonomy of Proctor et al. (2013): *acceptability, adoption, appropriateness, feasibility, fidelity, implementation cost, penetration, and sustainability*. Service-delivery outcomes include access, coverage, continuity, quality, referral completion, and supply-chain performance; patient / clinical outcomes include blood-pressure and glycaemic control, retention, adherence, complications, and patient experience. Studies cited in each cell are **illustrative rather than exhaustive**; because most interventions were bundled, several studies contribute to more than one cluster.
