# Supplementary material for: Implementation strategies for integrating non-communicable disease care into primary healthcare settings in sub-Saharan Africa: A systematic review
Source: PLOS Glob Public Health. 2026 Sep 15;6(9):e0007062. doi: 10.1371/journal.pgph.0007062 (PMC13577421; doi:10.1371/journal.pgph.0007062)
Supplement: Supplementary file 9 [file pgph.0007062.s009.docx]

**Studies excluded at full-text screening**

| **Study (author, year)** | **Title** | **Journal (year)** | **Reason for exclusion** |
| --- | --- | --- | --- |
| **Ouma et al. (2025)** | Assessing the capacity of primary healthcare facilities and healthcare workers in managing diabetes and hypertension in Kisumu county, Kenya | BMC Health Serv Res (2025) | No implementation/integration strategy: capacity/readiness (WHO SARA) assessment; no strategy implemented or evaluated. |
| **Mbatia & Jenkins (2010)** | Development of a Mental Health Policy and System in Tanzania: An Integrated Approach to Achieve Equity | Psychiatric Services (2010) | No implementation/integration strategy: mental-health policy/system development, not NCD–PHC integration. |
| **Orji et al. (2024)** | Evaluating diabetes care in primary healthcare centers in Abuja, Nigeria: a cross-sectional formative assessment | BMC Primary Care (2024) | No implementation/integration strategy: WHO SARA readiness assessment; no strategy evaluated. |
| **Tapera & Nyakabau (2020)** | Limited knowledge and access to palliative care among women with cervical cancer: an opportunity for integrating oncology and palliative care in Zimbabwe | BMC Palliative Care (2020) | Not a PHC setting: secondary/tertiary facilities; integration discussed only as a future opportunity. |
| **Nketiah-Amponsah et al. (2025)** | Willingness to pay for digital care for NCD in Ghana: an exploratory study of three health facilities | BMC Health Serv Res (2025) | Not a PHC setting: secondary/tertiary/quaternary facilities; willingness-to-pay valuation, not a strategy. |
| **Noumi, Houngue & Lontsi (1999)** | Traditional medicines in primary health care: plants used for the treatment of hypertension in Bafia, Cameroon | Fitoterapia (1999) | No implementation/integration strategy: ethnobotanical survey. |
| **Orji et al. (2024) [preprint]** | Evaluation of Primary Healthcare Centers' Service Availability and Readiness for Implementing Diabetes Care in Abuja, Nigeria | Research Square preprint (2024) | Duplicate record (preprint of the BMC Primary Care 2024 paper); also no strategy evaluated. |
| **Kruk & Nowicki (2018)** | Effect of the physical activity program on the treatment of resistant hypertension in primary care | Prim Health Care Res Dev (2018) | Not conducted in SSA: study conducted in Poland. |
| **Goma et al. (2016)** | Indigenous Knowledge Systems for the treatment of Hypertension in Lusaka, Zambia: Perceptions, Knowledge and Practice | Med J Zambia (2016) | No implementation/integration strategy: traditional-medicine perceptions study. |
| **Flood et al. (2022)** | Integrating hypertension and diabetes management in primary health care settings: HEARTS as a tool | Rev Panam Salud Publica (2022) | Not conducted in SSA: implementation evidence from the Americas region only. |
| **Nyirenda et al. (2023)** | Acceptability and feasibility of tuberculosis and diabetes mellitus bidirectional screening and joint treatment services in Malawi | BMJ Open (2023) | Not a PHC setting (borderline): eight secondary-level hospitals; no PHC-level integration component. |
| **Siko & van Deventer (2017)** | Compliance with standard treatment guidelines in the management of hypertension, Potchefstroom, South Africa | S Afr Fam Pract (2017) | No implementation/integration strategy: clinical audit of guideline compliance. |
| **Mboweni (2024)** | Primary healthcare nurses' experiences in managing chronic diseases during COVID-19 in the North West province, South Africa | Afr J Prim Health Care Fam Med (2024) | No implementation/integration strategy: nurses' pandemic experiences, no strategy evaluated. |
| **Onwukwe & Omole (2012)** | Drug therapy, lifestyle modification and blood pressure control in a primary care facility, south of Johannesburg | S Afr Fam Pract (2012) | No implementation/integration strategy: clinical audit of BP control. |
| **Muula (2021)** | Herbal Medicine, Diabetes Care at Primary Care Facilities and Prevention of Vertical HIV Transmission | Malawi Med J (2021) | Study design not eligible: editorial. |
| **Solomon et al. (2022)** | Addressing the Impact of NCDs and Injuries in Ethiopia: Findings and Recommendations from the Ethiopia NCDI Commission | Ethiop J Health Sci (2022) | No implementation/integration strategy: commission report/policy synthesis of epidemiological data. |
| **Ojo et al. (2016)** | An appraisal of blood pressure control and its determinants among patients with primary hypertension in a primary care setting in Western Nigeria | S Afr Fam Pract (2016) | No implementation/integration strategy: clinical audit of BP control and determinants. |
| **Shayo et al.** | Management of chronic conditions in resource-limited settings: multi-stakeholders' perception and experiences with receiving and providing integrated HIV, diabetes and hypertension services in Tanzania | BMC Health Services Research (2023) | Setting is a hospital-based integrated clinic (not explicitly community PHC). |
| **Aifah et al.** | Integration of a task strengthening strategy for hypertension management into HIV care in Nigeria: a cluster randomised controlled trial study protocol | Implementation Science (2021) | This is a study protocol, not a results paper. It does not report outcomes |
| **Maimela et al.** | The perceptions and perspectives of patients and health care providers on chronic diseases management in rural South Africa: a qualitative study | BMC Health Services Research (2015) | *No implementation strategy integrated* |
| **Musinguzi et al.** | An implementation science study to enhance cardiovascular disease prevention in Mukono and Buikwe districts in Uganda: a stepped-wedge design | BMC Health Services Research (2019) | This is a study protocol with no outcome results |
| **Teshome et al.** | Trained health extension workers correctly identify high blood pressure in rural districts of northwest Ethiopia: a diagnostic accuracy study | BMC Health Services Research (2022) | This is a diagnostic accuracy/validation study. It does not evaluate a full implementation programme |
| **Konlan et al.** | Development of Public Health Center-Based Culturally Tailored Hypertension Self-Care Intervention Among Adults in Rural Ghana | Public Health Nursing (2024/2025) | This is an intervention development paper, not a full implementation evaluation |
| **Angwenyi et al.** | Context matters: a qualitative study of the practicalities and dilemmas of delivering integrated chronic care within primary and secondary care settings in a rural Malawian district | BMC Family Practice (2020) | Study includes both primary and secondary care settings (Phalombe district) |
| **Nelissen et al.** | Pharmacy-based hypertension care employing mHealth in Lagos, Nigeria – a mixed methods feasibility study | BMC Health Services Research (2018) | Setting is private community pharmacies, not formal PHC clinics |
| **Engmann, Ampofo & Dowrick** | Integrating mental health and non-communicable disease care: A WONCA advocacy project report | African Journal of Primary Health Care & Family Medicine (2025) | This is a brief quality improvement (QI) project report (3 pages), not a full empirical research paper |
| **Ngassa Piotie et al.** | Designing an integrated, nurse-driven and home-based digital intervention to improve insulin management in under-resourced settings | Therapeutic Advances in Endocrinology and Metabolism (2021) | This is an intervention design/development paper (pre-implementation phase), not an outcomes study. |
| **Osehobo et al.** | Telemedicine promise for tomorrow: The role of video synchronous medical consultation in a primary health center (PHC) in a sub-Saharan Africa setting | Tropical Journal of Pharmaceutical Research (2024) | The telemedicine model involves USA-based rather than in-country physicians, raising questions… |
| **Onyenwenyi & Mchunu** | Primary health care workers' understanding and skills related to cervical cancer prevention in Sango PHC centre in south-western Nigeria: a qualitative study | Primary Health Care Research & Development (2019) | This is a needs assessment/situational analysis of PHC workers' knowledge and capacity for cervical cancer (NCD) screening using VIA, not an evaluation of a running implementation strategy |
| **Kengne et al. (CENHIP Investigators)** | Setting-up nurse-led pilot clinics for the management of non-communicable diseases at primary health care level in resource-limited settings of Africa | Pan African Medical Journal (2009) | Study describes the setup process rather than reporting final outcomes. |
| **Parham et al.** | A Modern Telecommunications Matrix for Cervical Cancer Prevention in Zambia | Journal of Lower Genital Tract Disease (2010) | This is a programme description/implementation report rather than a formal study with quantitative outcomes |
| **Lund et al. (PRIME Consortium)** | Integrating Mental Health into Primary Health Care: A Global Perspective (PRIME situation analysis across five LMICs) | PLOS ONE (2012) | This is a situational/baseline analysis, not an outcomes study |
| **Masupe et al.** | Integrated management of HIV/NCDs: knowledge, attitudes, and practices of health care workers in Gaborone, Botswana | African Health Sciences (2019) | *No implementation strategy integrated* |
| **Sorsdahl et al.** | Integration of mental health counselling into chronic disease services at the primary health care level: Formative research on dedicated versus designated strategies in the Western Cape, South Africa | Journal of Health Services Research & Policy (2020) | *Focus on views on the acceptability and feasibility of two models for integrating facility-based counsellor-delivered mental health counselling. No integration strategy* |
| **Opoku, Busse & Quentin** | Achieving Sustainability and Scale-Up of Mobile Health Noncommunicable Disease Interventions in Sub-Saharan Africa: Views of Policy Makers in Ghana | JMIR mHealth and uHealth | Participants are national policy makers, not frontline PHC workers, and the study focuses on sustainability/scale-up factors rather than evaluating a specific implemented mHealth programme |
| **Greve et al.** | Supporting Non-Communicable Disease Prevention Through a mHealth Application in Decentralized Healthcare Systems: Action Design Research in Eswatini | European Conference on Information Systems (ECIS 2020 Proceedings) | Published as a conference proceedings paper (ECIS 2020), not a peer-reviewed journal article |
| **Ntuli** | A Phenomenological Study of Primary Healthcare Nurses' Experiences in Assessing and Managing Diabetic Foot Complications | International Journal of Environmental Research and Public Health (2025) | This is a barriers/needs assessment study describing nurses' lived experiences and challenges with diabetic foot care at PHC level, not an evaluation of a running implementation strategy |
| **Williams et al.** | Diabetes Tuberculosis Care in Eswatini: A Qualitative Study of Opportunities and Recommendations for Effective Services Integration | International Journal of Public Health (2023) | Focuses on opportunities and recommendations |
| **Ibrahim et al.** | Cervical cancer screening in primary health care setting in Sudan: a comparative study of visual inspection with acetic acid and Pap smear | International Journal of Women's Health (2012) | This is primarily a diagnostic accuracy/validation study comparing VIA vs Pap smear in a primary health care setting. It does not evaluate a full programme implementation strategy |
| **Osetinsky et al.** | Layering and scaling up chronic non-communicable disease care on existing HIV care systems and acute care settings in Kenya: a cost and budget impact analysis | Journal of the International AIDS Society (2020) | This is a health economic analysis (cost and budget impact), not a clinical or implementation outcomes study |
| **Hickey et al.** | A prospective cohort study of the SEARCH integrated HIV/hypertension community health worker-led intervention in Kenya and Uganda | Journal of the International AIDS Society (2025) | This is a cohort sub-study of the SEARCH trial, reporting on the embedded CHW-facilitated hypertension telehealth intervention. |
| **Hickey et al.** | Effect of a patient-centered hypertension delivery strategy on all-cause mortality: Secondary analysis of SEARCH, a community-randomized trial in rural Kenya and Uganda | PLOS Medicine (2021) | No implementation strategy evaluated |
| **Ameh** | Evaluation of an integrated HIV and hypertension management model in rural South Africa: a mixed methods approach (PhD Review) | Global Health Action (2020) | This is a published summary/review of a PhD thesis rather than a standalone peer-reviewed empirical paper |
| **Nyaaba et al.** | Factors hindering hypertension control: perspectives of front-line health professionals in rural Ghana | Public Health (2020) | This is a barriers analysis study rather than an evaluation of a specific implementation strategy |
| **Badacho & Mahomed** | Facilitators and barriers to integration of noncommunicable diseases with HIV care at primary health care in Ethiopia: a qualitative analysis using CFIR | Frontiers in Public Health (2023) | *[Reason for exclusion to be added]* |
| **Muwanguzi et al.** | Barriers and facilitators to cognitive impairment screening among older adults with diabetes mellitus and hypertension by primary healthcare providers in rural Uganda | Frontiers in Research Health Sciences (2023) | Study focuses on integrating cognitive impairment screening (a mental health NCD) into PHC services for patients with DM and/or HTN — a specific NCD-PHC integration challenge. It is not about managing DM/HTN |
| **Alani et al.** | Lessons learned from implementing the Non-Communicable Diseases Kit in a humanitarian emergency: an operational evaluation in Sudan | BMJ Global Health (2023) | The WHO-NCD Kit is a health system intervention targeted at the PHC level in an emergency context. |
| **Hezagira et al.** | Three decades of community health workers in primary healthcare delivery in Rwanda: evolution, impact and policy lessons | BMJ Global Health (2025) | No implementation strategy |
| **Edwards et al.** | Improving cost-effectiveness of hypertension management at a community health centre | South African Medical Journal (1998) | *No implementation strategy* |
| **Agyen et al. (2025)** | The Role of Lifestyle Interventions in Reducing the Burden of Non-Communicable Diseases: Evidence from Community-Based Programmes | Ghana J Nurs Midwifery (2025) | Study was conducted in a regional hospital |
| **Haque et al. (2005)** | Barriers to initiating insulin therapy in patients with type 2 diabetes in public-sector primary health care centres in Cape Town | J Endocrinol Metab Diabetes S Afr (2005) | Barriers study; does not evaluate an integration strategy. |
| **Otieno et al. (2022)** | Assessing the Readiness to Provide Integrated Management of Cardiovascular Diseases and Type 2 Diabetes Mellitus in Kenya | Global Heart (2022) | readiness/capacity assessment, not an active implementation strategy. |
| **Rutayisire et al. (2020)** | Integration of Chronic Oncology Services in Noncommunicable Disease Clinic in Rural Rwanda | Annals of Global Health (2020) | NCD clinic at a district (secondary) hospital, not a PHC setting. |
| **Daniels et al. (2000)** | A structured record to implement the national guidelines for diabetes and hypertension care | S Afr Med J (2000) | *No implementation strategy* |
| **Tapela et al.** | Integrating noncommunicable disease services into primary health care, Botswana | Bull World Health Organ | Did not collect primary data |
| **Moyo-Chilufya et al. (2024)** | A Framework for Implementing Integrated HIV and Non-Communicable Disease Care at Primary Health Care Facilities in Southern Africa | Int J Integr Care (2024) | *Study design: A systematic review* |
| **Lebina et al. (2020)** | The cost and cost implications of implementing the integrated chronic disease management model in South Africa | PLOS ONE (2020) | *No implementation strategy* |
